# Financing intersectoral action for health: a systematic review of co-financing models

**DOI:** 10.1186/s12992-019-0513-7

**Published:** 2019-12-18

**Authors:** Finn McGuire, Lavanya Vijayasingham, Anna Vassall, Roy Small, Douglas Webb, Teresa Guthrie, Michelle Remme

**Affiliations:** 1 University of York (Centre for Health Economics), York, UK; 2 United Nations University-International Institute for Global Health, Kuala Lumpur, Malaysia; 3 London School of Hygiene and Tropical Medicine, (Centre for Health Economics in London (CHIL)), London, UK; 4 United Nations Development Programme (HIV, Health and Development Group), New York, USA; 5 Independent consultant, Cape Town, South Africa

**Keywords:** Health financing, Intersectoral, Co-financing, Pooled budgets, Social determinants of health

## Abstract

**Background:**

Addressing the social and other non-biological determinants of health largely depends on policies and programmes implemented outside the health sector. While there is growing evidence on the effectiveness of interventions that tackle these upstream determinants, the health sector does not typically prioritise them. From a health perspective, they may not be cost-effective because their non-health outcomes tend to be ignored. Non-health sectors may, in turn, undervalue interventions with important co-benefits for population health, given their focus on their own sectoral objectives. The societal value of win-win interventions with impacts on multiple development goals may, therefore, be under-valued and under-resourced, as a result of siloed resource allocation mechanisms. Pooling budgets across sectors could ensure the total multi-sectoral value of these interventions is captured, and sectors’ shared goals are achieved more efficiently. Under such a co-financing approach, the cost of interventions with multi-sectoral outcomes would be shared by benefiting sectors, stimulating mutually beneficial cross-sectoral investments. Leveraging funding in other sectors could off-set flat-lining global development assistance for health and optimise public spending. Although there have been experiments with such cross-sectoral co-financing in several settings, there has been limited analysis to examine these models, their performance and their institutional feasibility.

**Aim:**

This study aimed to identify and characterise cross-sectoral co-financing models, their operational modalities, effectiveness, and institutional enablers and barriers.

**Methods:**

We conducted a systematic review of peer-reviewed and grey literature, following PRISMA guidelines. Studies were included if data was provided on interventions funded across two or more sectors, or multiple budgets. Extracted data were categorised and qualitatively coded.

**Results:**

Of 2751 publications screened, 81 cases of co-financing were identified. Most were from high-income countries (93%), but six innovative models were found in Uganda, Brazil, El Salvador, Mozambique, Zambia, and Kenya that also included non-public and international payers. The highest number of cases involved the health (93%), social care (64%) and education (22%) sectors. Co-financing models were most often implemented with the intention of integrating services across sectors for defined target populations, although models were also found aimed at health promotion activities outside the health sector and cross-sectoral financial rewards. Interventions were either implemented and governed by a single sector or delivered in an integrated manner with cross-sectoral accountability. Resource constraints and political relevance emerged as key enablers of co-financing, while lack of clarity around the roles of different sectoral players and the objectives of the pooling were found to be barriers to success. Although rigorous impact or economic evaluations were scarce, positive process measures were frequently reported with some evidence suggesting co-financing contributed to improved outcomes.

**Conclusion:**

Co-financing remains in an exploratory phase, with diverse models having been implemented across sectors and settings. By incentivising intersectoral action on structural inequities and barriers to health interventions, such a novel financing mechanism could contribute to more effective engagement of non-health sectors; to efficiency gains in the financing of universal health coverage; and to simultaneously achieving health and other well-being related sustainable development goals.

## Introduction

Achievement of the Sustainable Development Goals (SDGs) will require substantial investment globally. The estimated price tag for the ‘health for all’ goal alone is USD 3.9 trillion for 75% of the world population [1, 2] while delivering on all the SDGs will need an *annual* investment of USD 3.9 trillion, with a current gap of USD 2.5 trillion [3, 4]. Amidst dynamics of escalating costs, growing populations and diminishing international development financing, the Addis Ababa Action Agenda on Financing for Development calls for accelerated and aligned mobilization of public, private, domestic and international financing; innovative financing mechanisms; and systemic change to harness the synergistic efficiency gains from investing across sectors and goals [5]. Indeed, the SDGs will not be achieved based on current financing trends and systems of planning, budgeting and service delivery that operate in sectoral siloes that do not value or prioritise development synergies [6, 7].

Despite strong calls for ‘whole-of-government’ approaches, ‘health-in-all-policies’, and ‘intersectoral action for health’, financing for health impact is still dominated by a sectoral approach reflecting a biomedical focus on proximal determinants of health [8]. Single sector financing is particularly problematic for the funding of structural interventions that address the social determinants of health, which have the potential to generate large health gains and synergies across the SDGs [9, 10]. For example, 50% of the global mortality reduction in children under-5 between 1990 and 2010 has been attributed to non-health sector investments, such as infrastructure development and expanding access to education [11]. Similarly, programmes and policies that increase gender equality (including gender-based violence prevention, economic empowerment for women, interventions to keep adolescent girls in school and transform unequal gender norms) have also been found to reduce disease risk, increase health service uptake and significantly improve health outcomes [12, 13].

However, health sectors rarely invest substantially in these intersectoral interventions, partly as a result of the prevailing narrow approaches to evaluating investment value, which often excludes the consideration of non-health costs and impacts [14]. Similar paradigms in other sectors may also undervalue health co-benefits from non-health sector investments. In recent years, there has been increasing recognition that intersectoral investment analyses should be adopted where relevant for investment in health [15, 16]; and that governments need to provide the incentives, budgetary commitments, and sustainable mechanisms to support multisectoral collaboration [8, 17]. Countries across income levels are beginning to explore how best to institutionalise these, and the funding flows that result from them [6].

Inter-sectoral co-financing could be one of the funding instruments to enable intersectoral action and overcome the fragmentation and inefficiencies of silo budgeting [9]. Co-financing is defined as the joint financing of a programme or intervention by two or more budget holders that have different sectoral objectives to jointly achieve their separate goals more efficiently. In theory, this could mean increasing the resource envelope for health spending by pooling funds with non-health sectors and thus leveraging additional investment in health, as well as more efficient purchasing of health-producing interventions beyond the health system [18].

Co-financing has been implemented in a number of high-income countries, but there is limited evidence of its impact on costs, funding flows or health outcomes. McDaid & Park (2016) reviewed case studies of financing and budgeting mechanisms for intersectoral collaboration for health promotion between the health, education, social welfare, and labour sectors. The authors identified three principal financing mechanisms: discretionary earmarked funding, recurring delegated financing allocated to independent bodies, and joint budgeting [19]. The latter reflects a co-financing approach, as it involves joint budgeting between two or more sectors. Mason and colleagues (2015) reviewed evidence on integrated financing models, which they referred to as integrated resource mechanisms, between the health and social care sectors in eight high-income countries [20]. Both studies found examples of successful co-financing that uncovered unmet need and improved short-term health outcomes, but overall, they concluded that there is limited evidence to conclusively demonstrate that co-financing has maximised programme and policy impact or lowered costs to sectoral payers. Similarly, the body of literature on the health sector’s involvement in intersectoral action only minimally addresses its financing implications [6, 21], including how budgeting and accounting arrangements are negotiated and implemented. More evidence is, therefore required to better understand which financing models can improve the uptake and sustainability of intersectoral collaboration across a more diverse range of settings.

To extend the reviews above, given the global remit of the SDGs and the range of sectors that can influence population health, we aim to review and synthesise evidence on co-financing arrangements beyond the health and social care sectors, and beyond high-income countries. In this article, we identify and characterise such cross-sectoral co-financing models, their operational modalities, effectiveness, and institutional enablers and barriers. We first present the typologies of co-financing models classified by benefits and financing mechanisms. We then present qualitative themes of barriers and enablers of uptake, implementation, and continuation of the co-financing approach, and discuss lessons learned and future implementation and research needs.

## Methods

### Definitions

Intersectoral co-financing (hereafter referred to as ‘co-financing’) cases were conceptualised according to two criteria. First, co-financing requires the joint commitment of resources towards an intervention or interventions by at least two budget holders. Resources can include financial or in-kind contributions. Second, the budget holders must have dissimilar programming objectives, or more specifically, they must be allocating their resources to achieve distinct end outcomes. These outcomes can be defined at a sectoral or sub-sectoral level (e.g. population health outcomes, such as lives saved or quality of life gained; or disease-specific outcomes, such as HIV infections averted). Two health sector budget holders combining their resources to achieve the same outcome (such as a Ministry of Health and an external donor) would not classify as co-financing given the shared objective.

The approach draws conceptually on health system financing, with its three distinct functions: revenue collection (to raise money for health); pooling of resources (to share the financial risks of paying for healthcare); and purchasing of services and interventions (to optimise the use of health resources) [22]. Although health financing focuses primarily on how to pay for healthcare services and public health interventions, co-financing focuses more on how to raise money for health outcomes across public sector payers, and then how to use those funds to purchase healthcare and non-health interventions that maximise health outcomes [23]. Clearly, what is considered ‘revenue collection’ for one sector (health, in this case) is a form of purchasing outcomes for the contributing sector. Moreover, the programmatic financial risk is shared across sectors, as no single-payer takes on the full cost of delivering the intervention/service, but this is quite distinct from the risk-pooling of individual health risk.

Co-financing can, therefore, involve sharing the revenue collection and/or purchasing functions across payers from different sectors. For the health sector, this would allow for non-health sector resources to be leveraged for health gain, and for strategic purchasing of non-health interventions with co-benefits. The types of financial mechanisms used to operationalise co-financing can be further sub-categorised, based on the typology summarised in Table 1 that is adapted from Mason et al. (2015) [20].

**Table 1:** Types of financial mechanisms for co-financing

Financial mechanism	Definition
Revenue collection	
1. Pooled funds	At least two budget holders make contributions to a single pool for spending on pre-agreed services or interventions. This can be done at various levels (national, regional, local) and accessed in different ways (i.e. grants or regular budgetary system).
2. Aligned budgets	Budget holders align resources, identify own contributions towards pre-specified common objectives. Joint monitoring of spending and performance, but management remains separate.
3. Structural integration	Full integration of cross-sector responsibilities, finances and resources under single management or a single organisation.
Purchasing	
1. Joint or lead commissioning	Separate budget holders jointly identify a need and agree on a set of objectives, then commission services and track outcomes. The commissioning itself can be done through a joint authority board or through one agency taking commissioning responsibility.
2. Cross-charging	The mechanism whereby a cross-sector financial penalty is incurred for the non-achievement of a pre-specified target. Cross-charging compensates sectors who incur an external cost from another sector’s poor performance.
3. Transfer payments	Sectoral budget holders make service revenue or capital contributions to bodies in other sectors to support additional services or interventions in this other sector.

*Adapted from Mason* et al. *(2015)* [20]

### Search strategy, article screening and inclusion

The review process was guided by the Preferred Reporting Items for Systematic Reviews and Meta-analyses (PRISMA) statement. A systematic search of peer-reviewed and grey literature was performed using a three-level process. First, sixteen electronic bibliographic academic databases were searched: Africa-wide Information, Applied Social Sciences Index & Abstracts, CINAHL Plus, EconLit, EMBASE, ERIC, Global Health, Health System Evidence, HMIC, IBSS, MEDLINE, PsycINFO, ScienceDirect, SCOPUS, Social Policy & Practise and Web of Science. Second, grey and policy literature was identified through a structured search of Google, Open Grey, the OECD iLibrary, the World Bank eLibrary, and ADOLEC Lit. Finally, reference snowballing or hand searches of included articles was used to identify any previously unidentified articles. No geographical or publication date constraints were applied. The search was limited to articles published in English before March 2018 (see additional file 2 for search strategy).

After removal of duplicates, each record (title and abstracts) was independently screened and categorised by two authors (FM and MR), and disagreements were resolved through discussions. Table 2 lists the inclusion and exclusion criteria for studies. Both qualitative and quantitative study methodologies and data were included – qualitative for establishing typologies and themes on the enablers and barriers, and quantitative to assess the effectiveness of co-financing models. Exclusion for poor methodological quality was not performed to provide a critique of current methods to inform future research design.

**Table 2:** Inclusion & exclusion criteria

Inclusion criteria	
• Studies describing a co-financing case (with or without an evaluation); • Co-financing between any two sectors or sub-sectors (i.e. no sectoral restrictions); • English language	
Exclusion criteria	
• Guidelines for how to implement co-financing; • Articles with insufficient information to adequately identify a co-financing case; • Commentaries or policy briefs mentioning co-financing (In these instances, primary studies were sourced); • Purely commercial relationships such as those between public sector actors and private sector contractors (e.g. Private Finance Initiatives or Public-Private Partnerships are not co-financing because the private entity’s profit objective is not a final outcome, it is an intermediate objective that the contractual arrangement aims to align to the ultimate public objective or outcome measure).	

Data from included articles were extracted using a customised tool (in Microsoft Excel) with domains including the description of the programme or intervention, country, and actors, administrative level of operation, payers, purpose of model, financial mechanisms, governance structures, study design, reported outcomes/effects, barriers and enablers of the co-financing cases. Where there were multiple publications on a specific co-financing case, these were included and used to extract the required information. Extracted data was used to develop a typology of co-financing models, drawing on the conceptual definition and financing mechanisms presented above.

Qualitative data on the enablers and barriers to uptake, implementation, and continuation of the co-financing approach was extracted when reported in a study. This was first coded using open unfocused coding, based on emergent in-vivo themes (non-a priori). Next, these open codes were grouped into higher-level categories and sub-themes, that were constructed based on an understanding of the text and context.

## Results

### Search results

**Fig. 1:**
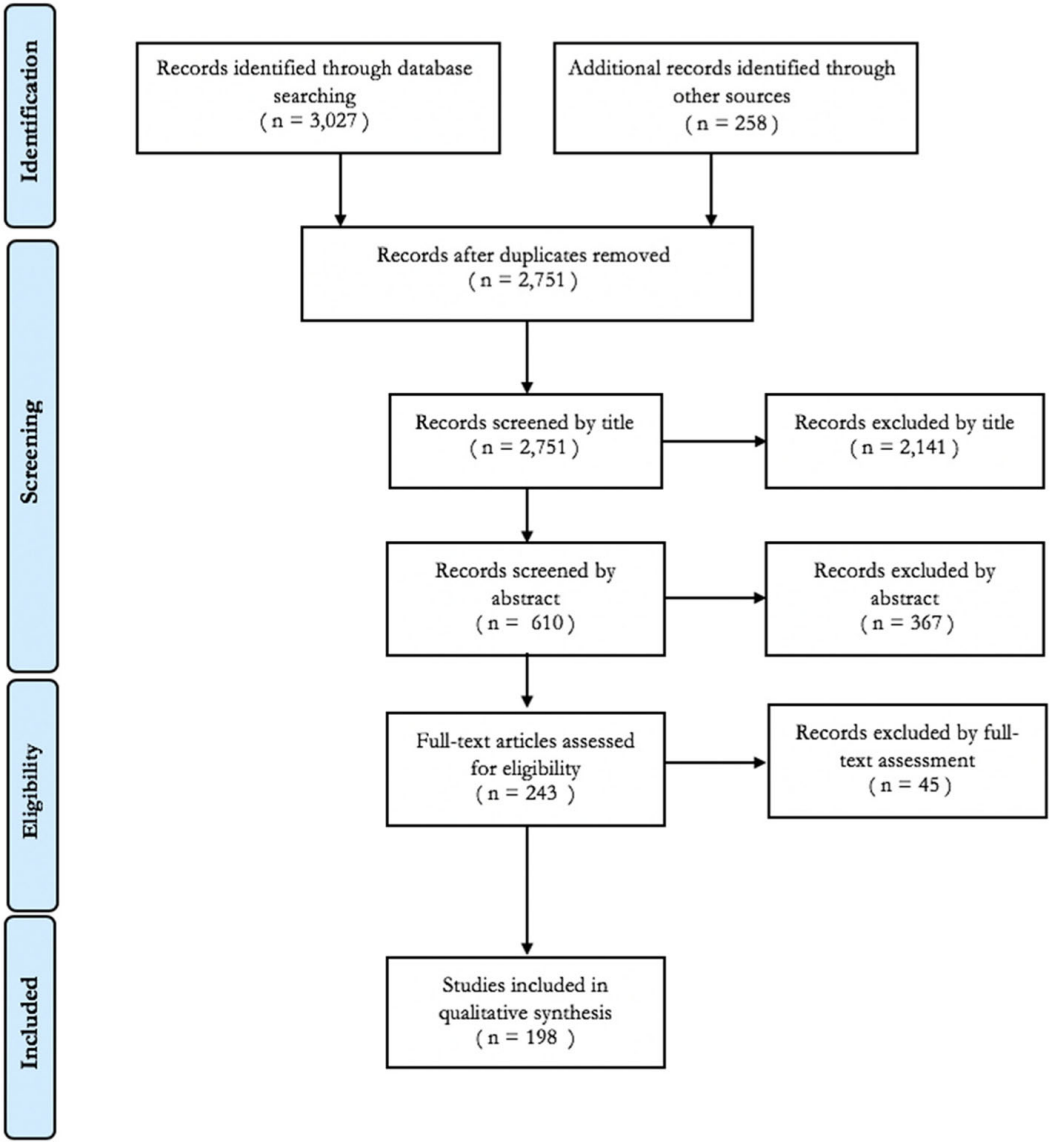
Article Screening and Inclusion based on PRISMA

The initial search identified 2751 publications (after duplicates removed). After screening, 198 publications were identified as eligible for inclusion (see Fig.1) and covered 81 separate implemented cases of co-financing. Most excluded cases involved integration or coordination without co-financing or co-financing between budget holders with identical objectives.

The included cases and articles reflected co-financing arrangements between the health, education, environment, agriculture, social care, housing, economic and labour sectors, with different end-objectives, or set of outcomes. Table 3 outlines the cases identified. More descriptions of included cases are provided as Additional file 1.

**Table 3:** Description of Cases

Total Included Cases		Total	Promotion Cases^a^	Integrative Cases^b^
		81	24	57
Country Development Status	High-Income Country	75	15	57
	Low-and-Middle-Income Country	6	9	0
Geographical Region	Europe	39	6	33
	North America	25	9	16
	South America	2	2	0
	Africa	4	4	0
	Asia	1	1	0
	Oceania	10	2	8
Co-financing Involvement of Sectors	Health	76	21	55
	Education	18	12	6
	Social Care	52	1	51
	Housing	5	3	2
	Justice	7	6	1
	Social services	5	4	1
	Agriculture	5	2	3
	Other	31	12	19
Co-financing partnership across sectors	Health + Social Care (only)	46	0	46
	Health+ Social Care +Other sectors	6	1	5
	Health + Education (only)	5	2	3
	Health + Education +Other Sectors (excluding social care)	10	9	1
	Health +Other Sectors (excluding social care and education)	3	3	0
	Health sector alone (as coordinators of intersectoral financing)	6	6	0
	Non-health sector	5	3	2
Administrative level of government	National	29	9	20
	State/ Regional/Local	52	15	37
Implementation Period	Pre-2000	27	3	24
	Post-2000	54	21	33

^a^Promotion Cases: single-sector investment in another sector to leverage resources and address upstream factors that affect its own sectoral outcomes

^b^Integrative Cases: integrated or coordinated service provision across sectors, often for a specific population group

More than half of the cases involved co-financing between the health and social care sectors (including social services and with other sectors), with the education sector as the next most frequent co-financing partner of the health sector. Only five cases did not involve the health sector (from the United States, England and New Zealand), but involved the education with social care or vocational rehabilitation sectors (*n* = 2), and the justice with vocational rehabilitation, housing and social care sectors.

### Typology of co-financing models and their financing mechanisms

The first characteristic that differentiated the co-financing cases identified was the objectives that they were trying to achieve. All co-financing cases found were broadly similar in their ultimate objective, that is to improve each participating sector’s outcomes and/or reduce their costs. However, we observed two distinct approaches for how the models would allow them to do so, which we define as ‘integrative’ co-financing and ‘promotion’ co-financing cases. Integrative models engaged in co-financing to integrate or better coordinate service provision across sectors, often for a specific population group. They tended to involve sectors with regular simultaneous or consecutive service provision. Most commonly, this involved financing the coordinated provision of health and social care services for a pre-defined population. For instance, integrated health and social care commissioning were introduced in Scotland in 2014 in response to the recognition of the importance of the social care sector in stemming rising expenditure in the health care sector [24–28]. Financial pressure on the health care system was predicted to worsen with the elderly population projected to increase by 85% between 2014 and 2039 while 34% of the household include at least one individual suffering from a chronic health problem or disability. The aim of the integration is to provide high-quality care and joined-up services that support people to stay in their homes. Similarly, the System for Integrated Care for Older Persons (SIPA) case implemented in Canada was aimed at community-based elderly individuals who are at-risk of requiring institutionalisation and frequent access to acute health care services [29–31]. The objective of the programme was to meet the health and social care needs of individuals in an integrated manner, through multidisciplinary team service provision.

Promotion models involved one sector investing in another sector, and leveraging its resources, to address upstream factors that affect its own sectoral outcomes. These were often models for health promotion. For example, the Prince Edward Island province in Canada, recognising the breadth of the factors that influence health, designed an explicit financial and governance mechanism to facilitate cross-sectoral action [32–34]. This mechanism included several sectors, i.e. health, education, housing, social security, employment, justice and city planning). Two promotion cases in Kenya and Zambia focused on the school-based provision of health services, specifically deworming, which involved the health and education sectors [35–41]. The education sector contributed resources to the intervention because of the recognition of the promotional effect of good health on education outcomes.

Unlike integrative models, promotion co-financing cases tended to involve sectors with less service overlap. Broadly speaking, integrative co-financing was population-centric, while promotion co-financing was more intervention-centric. The tables presenting the co-financing cases identified are disaggregated by co-financing typology and included as supplementary material.

The financial mechanisms that were used to operationalise these co-financing cases are summarised in [Table 4. Most of the integrative cases involved sub-national pooling of budgets, whereas most promotion cases were transfer payments or grants from a health payer, based on intersectoral project proposals. It should be noted that while the co-financing cases have been broadly categorised based on their primary function, several cases combined both revenue collection and purchasing functions. For example, the North-West London Integrated Care Pilot utilised lead commissioning, in tandem with the additional alignment of financial incentives whereby participating organisations agreed to share any savings that materialised from the pilot for joint re-investment [82, 94, 95].

**Table 4:** Description of financial mechanisms used to implement the co-financing approach

Financial Mechanism	Description	Review findings: number of cases and examples	
		Integrative cases	Promotion cases
Revenue collection			
1. a) National pooled budget	Pooled budgets were established at national level, with decentralised bodies developing plans for use of the funds related to local objectives and priorities. The aims of the individual decentralised cases were diverse and locally variable. Some consisted of braiding financing streams, whereby funds were not fully integrated, and it was possible to trace the source of each expenditure, while other included blended financing streams, whereby the money in the pool lost its sectoral source identity.	6 cases • Reshaping Care for Older People (Scotland) [24–28] • Children’s Trust Pathfinders (England) [42] • Better Care Fund (England) [43–48]	No case identified
1. b) Sub-national pooled budgets	This mechanism was often enabled through national legislation permitting voluntary budget pooling by local government across sectors and the joint commissioning of multi-sectoral services. Budget holders’ contributions were identified from the outset and expenditures were planned from inception in some cases, and not in others to allow flexibility in how the funds were used.	26 cases • Health Flexibilities Act Section 75 for Clinical commissioning groups and local authorities (England) [47, 49] • SOCSAM (Sweden)-legislation that enabled social insurance, social services and health care services to be jointly co-financed between government and municipals [50–52] • Australian Capital Territory (ACT) Coordinated Care trial (Australia) [30, 53–55] • System of Integrated Care for Older Persons (Canada) [29–31, 56]	5 cases • Justice Sector Fund (New Zealand) [57–59] • Prince Edward Island (Canada) [32–34] • Ceará Multi-Sector Social Inclusion Development Program (Brazil) [60–67]
2. a) Aligned budgets	Aligned budget tended to be adopted where partnerships were yet to mature or where there was a concern that partners would be over-cautious or under-fund pooled budgets. whereby numerous ministries contributed to activities within the programme but the management and accountability for the resourcing remained entirely separate. There were often no statutory hindrances or restrictions.	2 cases • Financial Coordination of Rehabilitation Measures Act (FINSAM) (Sweden) [68–70] • Community Health Partnerships (Scotland) [71, 72]	3 cases • Programme for the Modernisation of Agriculture (Uganda) [73–76] • Interagency Programme for the Empowerment of Adolescent Girls (El Salvador) [77] • Pathfinder’s Geração Biz (“busy generation”) Programme for sexual and reproductive health and HIV (Mozambique) [78–81]
2. b) In-kind support	Sectors contributed non-financial resources (e.g. human resources, infrastructure, and technology) towards the joint provision of an intervention or programme with a shared objective. This mechanism was often used in circumstances where sectors were constrained by very limited financial resources, limited financial autonomy or in-kind support was more beneficial to service delivery than additional financial resources	No case identified	2 cases • School Health and Nutrition (Zambia) [35–37] • National School-Based Deworming Programme (Kenya) [38–41]
3. Structural integration	Full organisational and budgetary integration of cross sector responsibilities into a single organisation.	8 cases • Care Trusts at local area level with full responsibility for the pooled budget and purchasing for health and social care (England) [47, 73, 82–87] • Health and Social Care Boards commissioning services (Northern Ireland) [24, 88, 89]	No case identified
Purchasing			
4. Joint/lead commissioning	Joint commissioning was undertaken with and without pooled budgets. The commissioning of services with pooled budgets could be undertaken through lead commissioning whereby one agency was delegated authority for purchasing services across the jurisdiction of all sectors contributing to the pool. Otherwise, a joint authority was established with representatives of all pool contributors, managing the pool on behalf of partners, through agreed delegation arrangements.	6 cases • Contra Costa County Community Services Department coordinated funds for early education (USA) [90–93] • North West London Integrated Care Pilot (England) [82, 94–99] • The Home Loans Equipment Centre (HLEC) (England) [80, 94, 100]	No case identified
5. Cross-charging	Cross-charging was implemented as a form of Pigouvian taxation, where a sector’s performance incurred an externality on another sector. It often involved little to no integration of functions, organisations or services.	3 cases • National Health Service (NHS) mandatory daily penalties between local authorities and health care providers for delayed hospital discharges due to delays in social services (England) [101–105] • Denmark cross-charging (Denmark) [106] • ADEL reform (National Reform of Elderly Care) (Sweden) [105]	No case identified
6. Transfer payments	These mainly involved a grant-making mechanism set up by one payer that required intersectoral proposals. They were different from pooled budgets requiring grant applications in that the financial allocation originated from a single sector but was exclusively accessible for intersectoral action. Such grants were often disbursed by health promotion foundations.	No case identified	8 cases identified • Road Safety Partnership Grant (England) [65–67] • New York City Childhood Asthma Initiative (USA) [107, 108]; • National Development Programme for Social Welfare and Health Care Theme (Finland) [109–114]

Table 3 also categorises case descriptions according to the financing typologies. In promotion cases, co-financing between health, education and other sectors (excluding social care) were most common, and arrangements where the health sector as lone coordinators (*n* = 6; mostly providers of intersectoral grants) was only apparent in promotion cases. All cases from Africa, Asia, and South America were promotion-based, occurred after the year 2000, and the six cases from Africa and South America all involved the health and education sector (mostly targeting children and adolescent populations) with two of these cases also including the agriculture sector. The single Asian case (from South Korea) only included the health sector as a coordinating body of earmarked sin tax that was distributed to intersectoral partners [115, 116]. Co-financing in Africa and South America also involved financing from international donor and development agencies, such as the World Bank, UNDP, the Japanese International Cooperation Agency (JICA) and the Gates Foundation.

In addition, the cases could be further distinguished according to whether the financing first flowed through sectors or not. *Ex ante* co-financing were cases where funds were pooled prior to allocation across different sectors (e.g. national pooled budgets with grant applications, or sectoral grant mechanisms requiring intersectoral proposals) while *ex post* co-financing occurred when sectors that had already been allocated sectoral funds from a central authority subsequently dedicated those funds to co-financing arrangements. Although certain mechanisms, such as cross-charging, only fall under the post-allocation characterisation; others like pooled budgets were created before or after funds were allocated at the sectoral level. It was not always possible to identify the origin of the funds dedicated in co-financing cases.

### Evidence on impact from co-financing cases

#### Evaluation of study designs

Table 5 outlines the types of methodologies and study designs used in the included articles. These studies focussed evaluating the interventions and programme areas that were co-financed, rather than on the co-financing mechanism itself. Most cases had more than one evaluation and used more than one methodology. Case study design and qualitative methodologies were most dominant, which included stakeholder interviews, focus groups, and document review. Many cases identified broad and subjective outcomes which were neither sufficiently specific nor measurable for the use of quantitative approaches. The second most common evaluation design was uncontrolled trend analysis, frequently using routinely collected administrative data. Trend analysis was often undertaken alongside a qualitative evaluation, with less focus on the quantifiable impact of the co-financing mechanism. Few studies (15) utilised more rigorous evaluation methodologies, such as quasi-experimental evaluations utilising natural experiments, namely difference-in-difference, and matching methodologies. A smaller number of cases (7) set out to purposefully establish the causal effects through randomised controlled trials. No associated evaluations were identified for 26 co-financing cases, often citing a lack of financial resources and the scale of the evaluation challenge given the complexity of evaluating multifaceted system-level policies. It is important to note that implemented cases of co-financing were nearly always multifaceted system-level interventions, characterised by a number of governance, financial and regulatory components. None of the evaluations attempted to isolate the impact of the component parts of the co-financing cases, such as financial integration compared to the governance integration.

**Table 5:** Evaluation methodologies of co-financing cases

Evaluation methodology	No. of cases
Qualitative (including interviews, focus groups, document review, questionnaires, workshops, etc.)	37
Randomised controlled trials (RCTs)	7
Quasi-experimental	15
(Uncontrolled) trend analysis	24
No evaluation identified	26

*If an evaluation used more than one methodology, as outlined in the table, these were recorded as separate evaluations e.g. the evaluation of Children’s Trust Pathfinders utilised both trend analysis and qualitative evaluations, therefore, both were captured in the above table

#### Reported Health outcomes

Health outcomes assessed in the identified cases with the health sector included mortality, morbidity, health-related quality of life, service utilisation (inpatient/outpatient admissions, delayed discharge, nursing home admission, etc.), and satisfaction measures (provider and service user) Table 6 outlines the types of outcomes reported within the included cases.

**Table 6:** Health Outcomes measures by model type

Outcome	Integrative co-financing	Promotion co-financing
Health outcome (health-related quality of life, mortality)	20 of 57 cases • 14 evaluations reported no effect • 3 reported a positive effect • 1 reported a mixed effect • 2 reported a negative effect	7 of 24 cases • 7 evaluations reported positive effects
Health service utilisation (delayed discharge, hospital admissions, readmissions, nursing home admission etc.)	19 of 57 cases • 4 report no effect • 9 report reduction in health care utilisation/cost • 5 report mixed effects • 1 report increase in health care utilisation/cost	4 of 24 cases • 4 cases reported positive effects
Non-health outcomes (school completion, grade average, criminal offending rates, individuals housed, average wage, employment rate, etc.)	4 of 57 cases • 3 cases reported positive effects • 1 case reported no effect	8 of 24 cases 8 cases reported positive effects

Of the **57** integrative co-financing cases, only **20** attempted to evaluate the effect on health outcomes. Most of these used quasi-experimental or experimental methodologies **(14/20).** Ten of these evaluations reported no effect of the co-financing on health outcomes; three cases reported a positive effect; **one** case each reported mixed and negative effects. Predictably, many of the evaluations that assessed the effect on health outcomes also examined the effect on service utilisation **(13/20)**. Less than half of the cases that reported on health service utilisation found a positive effect in terms of reduced health care utilisation and cost.

Fewer promotion co-financing models examined the effects on health outcomes **(7/24)** and service utilisation **(4/24)**. The evaluation of a majority of promotion cases was undertaken through qualitative or (uncontrolled) trend analysis. A minority of promotion cases (3/24) undertook a (quasi-) experimental evaluation. This may be partly explained by fewer cases involving the health or social care sectors. All of these cases reported some degree of a positive effect from the co-financing approach.

#### Reported non-health outcomes

Only 12 of 81 cases reported performance on non-health outcomes. Other sectoral outcomes reported for integrative co-financing cases included educational attainment and school completion rates, housing, employment and wage rates. Promotion co-financing cases reported only positive effects for the following non-health outcomes: cognitive test scores, average grades, access to special education and reduced illiteracy; reduced contact time with the police and police call-outs; access to supportive permanent housing; households with water connections; improved children’s diets; increased farmers’ income and livestock production. Two of the six cases from LMICs (Uganda and Brazil) included evaluations of health, education, economic development, agricultural, water and sanitation outcomes. In Uganda, evaluations of the Programme for the Modernisation of Agriculture indicated that there were economic returns from advisory services, where farmers perceived that their livestock increased, their yields were either constant or increased and overall felt that community wealth was improving [73–76]. In Brazil, the Ceará Multi-Sector Social Inclusion Development Programme contributed to a significant reduction in illiteracy within the population over the age of 15 years [60–67].

Table 7 illustrates that a significant number of payers participated in co-financing without any collection of relevant indicators to attempt to attribute evidence of sectoral benefit. There are also a number of cases in which an evaluation took place, but relevant outcome data for at least one participating payer were not collected.

**Table 7:** Assessment of relevant cross-sectoral outcomes

Sector payer	Data on relevant outcomes	
	Collected	Not collected
Health	31	23
Social Care	11	28
Education	4	13
Justice	2	7
Housing	2	2
Other (agriculture, vocational rehabilitation, environment, transport etc.)	2	7

*Sector outcomes of interest are, to a degree, subjective, and at times not easily assigned to any single sector, an attempt at classification has been made to classify under sectors deemed most relevant

### Barriers and enablers to uptake, implementation and continuation of co-financing

The core themes identified across both barriers and enablers included: (1) conceptual buy-in; (2) model planning, design, implementation and framing; (3) organizational capacity, resources and time horizons; (4) relational factors and organizational culture; (5) finance and accounting practices; and (6) evidence, monitoring and accountability. Table 8 outlines the synthesised categories and themes of reported barriers and enablers, along with examples of open codes that were grouped into the thematic categories.

**Table 8:** Themes and Open Codes of Barriers and Enablers to Uptake, Implementation and Continuation of Co-financing Models

Example of Open Codes for Barriers	Sub Categories of Barriers	Theme	Sub-Categories of Enablers	Example of Open Codes for Enablers
• perceived risk, • ambiguities, • unclear timeframes to realise positive impact, • concerns over likely power shifts, • concerns over expansion of duties without matched increase in capacity, • perceived sense of position threat, • streamlining of functions leading to job loss, • lack of buy-in from actors across levels, • fear of impact on branding and position, • unsupportive public/client groups • Perceived underperformance of programme	Actor resistance due to perceived risk, ambiguities and threats	Conceptual Buy-In	Favourable political climate, client, actor and public support	• Recognition of need for change • effective incentives and perceived value, • limited resource as opportunity
• upstream-downstream discord-non-aligned prioritisation between administrative levels of government, • lack of consensus in negotiated details, • ambiguous terms, • inequitable funding arrangements, • lack of pre-defined responsibilities, • dissimilar shared purpose (operational, programmatic and of partnership), • (lack of) unity between leads, • ineffective change management, • unclear lines of authority	Unclear terms and unmatched partnership	Model Design, Planning Framing and Implementation	Effective planning	• Specific and outcome focussed framing in design and implementation, • extensive stakeholder consultation, • space for flexibility, • sustainability planning, • external facilitation and mediation
			Context level for implementation	• Actors were positioned to facilitate intersectoral coordination
• limited resources as obstacle, • differential IT infrastructure- hardware/Software, • lack of shared information sources, • turnover of key positions (operational) • hardware and software	Inadequate or incongruent resources	Organizational Resources and Capacity	Matched Partnership	• Matched partner resources - equal size, capacity, financial equity, • decision-making and implementation
• differences in pay scale, • different operational processes	Differences in human resources and ways of working		Adequate Expertise and Capacity	• Expertise of implementing team, • multidisciplinary capacity, • capacity to offset risk and uncertainties, • ability to be responsive to needs, • stability of key positions
• lack of leadership readiness, • no leadership buy in	Leadership		Leadership	• Strong leadership, • Prioritisation from leaders, • low turnover of leadership position
• timelines not sufficient to produce impact, • long-term sustainability to continue beyond pilot or single term	Time		Time	• Time to foster relationship and achieve impact
• no confidence in partners, • poor relationships, • different work culture/practices/processes, • strained communication, • unmatched prioritisation of co-financing between collaborators	Non-constructive relational and work dynamics	Relational and Organizational Culture	Established positive relational and work dynamics	• Extensive engagements, • effective relationships, • mutual trust, • culture of collaborative work, • history/record of collaborative work and partnership, progressive understanding of each other organization, culture, and practices, • joint-training and knowledge dissemination
• weak and subjective evidence, • access to data and confidentiality issues, • lack of common culture, record and practice for accountability, • different data reporting requirements	Insufficient result-focussed practices	Evidence, Output Data Monitoring and Evaluation	Set targets	• Creation of interagency performance targets
			Evidence of success	• Demonstrated success of pilot initiatives
• different accounting techniques, • reduced sense of financial flexibility, • rigidity in resource allocation, • rigid line-item accounting, • unanticipated rise in costs, or non-budgeted cost domains, • no matched change in accounting practice, • allocations based on historic trends-not current needs	Unmatched methods and capacity to adapt to needs	Finance and Accounting Practices	Financial control	• shared pre-negotiated control of funds

A critical theme for the implementation of co-financing models was the accounting, financial practices and organizational capacity to implement change. Co-financing often required the reprogramming of financial resources by at least one budget holder, which was difficult given its impact on vested interests and the legacy of competition within the public sector [117]. Furthermore, in the face of constrained budgets, several studies reported an understandable desire for public actors to behave conservatively, reverting to traditional core functions, and safeguarding resources. A lack of financial resources and high resource requirements of activities perceived as the core, such as acute health care, was commonly cited as a barrier for any or further engagement with co-financing. Additionally, rigid budgets and the lack of budgetary autonomy were stated as barriers to co-financing uptake and implementation [118]. Government ministries and departments often had mandates to provide certain services or received budgets ring-fenced for certain activities. The degree of autonomy over financial resources differed across levels of governance and level of fiscal decentralisation, with more flexibility reported at the decentralised level. Interestingly, limited financial resources were perceived as both a barrier to the implementation of co-financing and an enabler to getting cross-sectoral buy-in. Depending on the context, limited resources was discussed as a reason to pool resources with other sectors, and in separate cases, were also discussed as a limiting factor that prevented the implementation of co-financed programmes [43, 83, 119, 120].

Differential organizational capacity, resources, regulatory requirements and operational processes were also cited as barriers to implementation. The legalities of co-financing were found to be complex in some cases, given sectors’ different regulatory and accountability systems. For example, this was raised in relation to co-financing of adult health and social care in England, where health care provision is universal and free while social care is means-tested and any joint working must take this into account [43–48]. Relatedly, the theme of relational and organisational cultures emerged as key factor in the initiation, and implementation of co-financing, which was enabled by trust and matched partnerships. From the cases identified, it was clear that certain sectors were more amenable to co-financing, given historical connections of working together. Leveraging these historical relationships could be an enabler to establishing co-financing arrangements. It could be argued there is an inverse relationship between past integrated activities and the strength of the rationale required for adopting co-financing. Additionally, failures from past programmes were also observed as catalysts to try new approaches.

The importance of data, monitoring and accountability frameworks was highlighted across studies, as a means to foster initial and ongoing conceptual buy-in amongst resistant actors and to track benefits from co-financing for the partners involved. A key theme on barriers to uptake was uncertainty around the value of co-financing in practice, due to a lack of evidence, and uncertainty around the objective and scope of the co-financing approach, particularly if imposed in a top-down manner. Conversely, a key enabler for co-financing continuation was demonstrated success from co-financing pilots [121], which could lead to the replication or scaling up of the model. For instance, the adoption of a National School Health Policy in Zambia originated from a pilot programme with rigorous evaluation and positive impacts, which encouraged the Ministries of Health and Education, among others, to formalise the programme and scale up nationally [35]. Additionally, the creation of interagency performance targets was shown to be a potential catalyst for the initiation of co-financing arrangements [121]. By holding several agencies collectively responsible for achieving pre-specified targets, governments incentivised intersectoral partnerships in New Zealand [57].

## Discussion

In this article, we identify and examine cases of co-financing between the health and/or other non-health sectors, with a focus on their objectives, financial mechanisms, reported impact, institutional barriers and enablers. Findings suggest that co-financing of programmes or interventions by multiple benefiting sectors has been implemented in a range of settings, in various ways and with varying degrees of success. We identify two dominant types of co-financing models: integrative models that mobilise resources and fund integrated service provision across sectors (an extension of Mason and colleagues’ work) [20]; and promotion models that fund programmes that address upstream factors to promote a downstream sectoral objective. While integrative models were largely operationalised through sub-national pooled budgets with some form of joint or lead commissioning and were most common in high-income country settings, promotion models were more diverse and tended to use aligned budgets or grant modalities to fund intersectoral projects.

As highlighted in previous literature in this area, the current body of evidence on the practice of co-financing is still weak by virtue of the success metrics and evaluation methodologies used, as well as the level and scale of implementation [19, 20]. The relatively small number of identified co-financing cases also reflects both the general difficulty of undertaking inter-sectoral programming, and the specific challenges of engaging inter-sectoral financing mechanisms. In the cases reviewed, co-financing arrangements included a complex and customised mixture of governance, monitoring and evaluation and planning. Co-financing did not always lead to the efficiency gains that are theoretically possible, or this was not assessed, suggesting that further focus on impact, costs and optimising implementation is required.

Nevertheless, this updated and extended evidence on co-financing implementation demonstrates that it is institutionally feasible in a range of settings and sectors, including in low and middle-income countries, and additional sectors beyond health and social care. The diversity of cases indicates that there is no ‘blueprint’, nor a single set of contextual characteristics necessary to support a co-financing approach.

Our thematic synthesis on enabling factors and barriers to uptake, implementation and continuation of co-financing aligns with existing knowledge on intersectoral action involving the health sector, including the need for strong leadership, aligned formal and informal processes, individual and organizational trust, and credible accountability mechanisms [21, 122, 123]. However, evidence on intersectoral action for health often neglects the technical aspects of financing arrangements, and the specific requirements for them to work, such as the need for balanced financial contributions from partners, and budgetary autonomy and flexibility. Specific skills are required in the development stage of such arrangements, including negotiation, resource mobilisation, effective communication and public financial management.

Context and policy architecture were important in each of the cases reviewed. For public sector payers, the macro-fiscal environment, and specifically how public financial systems were organized, such as fiscal centralisation or decentralisation across national/federal or state/district governments for various sectors and public functions, influenced how co-financing was then organized and implemented. Co-financing may be more difficult in more centralised policy environments, particularly those without a top-down directive to co-finance or without enabling policy precedence and infrastructure.

Compared to pursuing co-financing ‘from scratch’, there was some indication that co-financing may be most feasible and impactful where enablers – including political will at the requisite level, an evaluated and successful pilot/programme, a multisectoral plan or performance targets with multi-sectoral accountability, an intersectoral governance structure, accountability and monitoring capacities – are already in place and where efficiency is a more central consideration. Projects with existing (external) funding, particularly funding which incentivizes innovation, may increase policymakers’ and budget holders’ willingness to experiment.

Another observation was that more formal evaluation structures for projects were implemented at the national level. Potentially due to larger amount of financial investments involved, nationally implemented cases of co-financing tended to document outcomes more comprehensively. Nevertheless, nearly two-thirds of cases were at state, district or local levels of context. One significant observation in sub-national, state or local cases, was that co-financing was largely engaged in voluntarily through a joint recognition of the benefits of a cooperative approach. In both national and sub-national co-financing, it was common that only the outcomes and targets of a single (usually the dominant or driving) sector was tracked and evaluated. This suggests that evidence of multi-sectoral gains was not the main justification for co-financing; or that there was sufficient trust that these benefits were being realised.

### Study limitations

This endeavour to systematically assess the implementation of co-financing is not without its limitations. First, there is a debate about what constitutes a case of co-financing and the boundaries of the concept. We have applied our rational conceptualisation of co-financing to categorise cases, but the reality is that co-financing can be framed and achieved in multiple ways; this can be politically advantageous, but it also makes objective classification difficult. Second, our approach to data extraction and synthesis relied on the clear identification of the objectives of payers. While theoretically, it is simple to distinguish organisations with a health objective from those with an education objective, in reality, this distinction is not always clear. Relatedly, with growing recognition of the value of multi-sectoral approaches that address upstream determinants of health (and other social outcomes), organisations are frequently expanding their operational space. Third, whereas McDaid and Park classified fiscal incentives (such as tax breaks) as joint budgeting [19], we excluded these cases, since resources coming directly from the Ministry of Finance are yet to have a sector-specific objective assigned to them. Finally, given the international scope of the cases identified, the English language restriction in the search may have excluded several non-English reports in the synthesis process. In the snowball retrieval of literature, a small number of non-English texts were identified for included cases from Sweden and Brazil (e.g. SOCSAM, Ceará Multi-Sector Social Inclusion Development Programme), but these were not analysed.

More research is needed to establish a credible evidence base on the impact of co-financing. Evaluation was frequently constrained by several factors. First, the systematic review revealed the lack of documented co-financing cases and a risk of publication bias. Secondly, of the small number of implemented cases, many did not engage in any formal evaluation. Thirdly, when there were evaluations, their study design often lacked the rigour required to make conclusive statements about the success or failure of the case and convincingly attribute observed changes to the implementation of co-financing. Future implementers of co-financing should consider more rigorous design and dissemination of impact evaluations, economic evaluations, and implementation research.

### Considerations for Intersectoral co-financing in the context of the SDGs

Given the emphasis placed on synergies between goals and targets in the SDG agenda, co-financing could be an innovative financing mechanism to help sectors work together to more efficiently achieve their respective SDG goals in a coordinated manner. From our analysis, we find that the health, education and social care sector are established intersectoral partners for co-financing, based on extensive historical relationships and interactions in many high-income countries. Although no attempts were made to prioritise any sectors in the search strategy, publication bias cannot be ruled out, where some sectors may be more likely to conduct and publish evaluations. It is also possible that health may have been the most prominent sector in the identified studies, because it is relatively more advanced in analysing and addressing upstream determinants. The health and social sectors could qualify as ‘first-movers’ in adopting the principles of co-financing, given the clear overlaps and targets for coordinated service delivery.

A number of clear opportunities for SDG synergies between sectors have been identified in literature [10, 124, 125], including from sectors that do not have a history of coordination or collaboration. In many circumstances, interested actors may still need to ‘make a case’ to engage in new financial relationships across sectors, but could benefit from framing co-financing as an opportunity to advance prominent health agendas.

Almost all of the cases from LMIC involved domestic public sector actors, in collaboration with international donors. Only one case appeared to be fully driven by public sector ministries (Mozambique). Many public funders in LMICs are faced with the task of enhancing domestic spending, optimizing the fiscal space for health amongst other public goods, and reducing dependence on out-of-pocket expenditures and overseas development assistance. Leveraging external financing to catalyse the development of innovative context-adapted models can create potential ways to expand allocations for health or other sectoral spending.

The inclusion of co-financing in national and local planning and financing frameworks for the SDGs, including those agreed between national authorities and development partners, could therefore be an opportunity [23]. The United Nations Development Programme (UNDP) has developed the Mainstreaming Acceleration and Policy Support (‘MAPS’) approach to SDG implementation at country-level to support countries to identify interventions with high-impact across sectors. The MAPS approach aims to align UN support to member states in making trade-offs in relation to SDG targets, and in identifying policies and interventions that have impact and ‘reach’ across multiple targets. Co-financing could be a potential fiscal instrument to realise efficiency gains, should the MAPS-generated political will extend to reformulating budgetary practices. Also, UNDP’s network of 60 country-based ‘accelerator labs’ (in development) aims to identify challenge-solution pairs through iterative learning and experimentation, for which co-financing could have potential for ‘SDG accelerator financing’.

At an international level, multi-lateral organisations and global donor agencies that have health and cross-sectoral mandates could also play a role in breaking the dominant siloed approach to global health financing, and driving co-financing initiatives, experimentation and research forward. For instance, The Global Action Plan for Healthy Lives and Well-being [126], which is a joint initiative of 12 global health institutions, offers a window of opportunity to adopt co-financing approaches for greater efficiency and joint impact. The plan includes the acceleration of sustainable financing goals, with sub-aims to increase domestic spending on health and the use of national fiscal and public financial management reform, efficient investments, and innovative joint financing strategies including multi-donor trust funds to achieve these outcomes.

At national levels, influential political champions (individuals and sectors) drive the uptake and integration of co-financing in national policies and strategies [127]. The political dimensions of co-financing appear as, if not more, important than the technical details. The few national level pooled funding arrangements identified focused on issues or population groups (e.g. older persons, children and better care) with relatively broad political buy-in and visibility.

Notable barriers to co-financing uptake and continuation are the lack of supporting evidence, ambiguous risk profiles and capacity to organize and implement. The SDGs have placed a heavy emphasis on measurable targets, and to determine whether co-financing could be an evidence-based approach to financing the SDGs, there is need for a body of evidence on its benefits, trade-offs or limitations. Co-financing does not, by definition, increase aggregate efficiency or lead to cost-savings. In fact, Mason and colleagues suggest that if integrated care and funds are successful, they are more likely to uncover unmet need and lead to increased costs, as well as improved health. This is also to be expected in the context of expanding universal health coverage. Cost-effectiveness and value for money may therefore be more relevant considerations than cost-savings. The potential gains that are likely to be achieved through using a co-financing approach, needs to be assessed alongside the transaction costs and likelihood of success of initiatives.

## Conclusions

The urgent need to collaborate effectively, ensure coherence and increase resources for health within and beyond the sector, is well-established. The health-in-all-policies principle is at the heart of contemporary policy paradigms and calls to action [128], and in many cases this may require financing mechanisms and incentives that enable intersectoral action. There has been little implementation guidance on how to operationalise these calls, at least at a level of mobilizing intersectoral resources and strategically purchasing intersectoral interventions. The findings from this review contribute to this limited body of implementation literature, but there is need for more evidence and systematic documentation and learning, particularly from low and middle-income countries.

The 2030 Agenda for Sustainable Development has the potential to be an impetus for more and better resourced intersectoral action. Achieving the 17 goals and 169 targets of the inter-linked SDGs, with finite resources, requires greater attention to value for money, stronger pursuit of innovation and deepened partnerships. New ways of collaborating and aligning policies and investments are likely to be tested and negotiated. In this context co-financing may be a tool to overcome barriers such as perceived risk and ambiguities, rigid budgetary structures and guidelines, and lack of historical collaboration between concerned sectors. While available literature and lessons on co-financing cases is limited, it is growing, and provides formative operational insights on how current co-financing models are implemented and where they have produced impact in practice.

## Supplementary information

**Additional file 1.** Case Descriptions, including country, financing mechanisms, outcomes data

**Additional file 2.** Search String used in 3 searches: Ovid Search terms for EconLit, MEDLINE, EMBASE, PsycINFO, HMIC, Global Health, Social Policy & Practise. Scopus, Open Grey

## Data Availability

Additional data (on individual cases and search strategy) is provided.

## Abbreviations

PRISMA: Preferred Reporting Items for Systematic Reviews and Meta-analyses statement
SDG: Sustainable Development Goals
HIV: Human Immunodeficiency Virus

## References

[1] Mohammed AJ, Ghebreyesus TA (2018). Healthy living, well-being and the sustainable development goals. Bull World Health Organ.

[2] Stenberg K, Hanssen O, Edejer TT-T, Bertram M, Brindley C, Meshreky A (2017). Financing transformative health systems towards achievement of the health sustainable development goals: a model for projected resource needs in 67 low-income and middle-income countries. Lancet Glob Health.

[3] OECD. The imperative of blended finance. Making Blended Finance Work for the Sustainable Development Goals: OECD; 2018. p. 37–46. Available from: https://www.oecd-ilibrary.org/development/making-blended-finance-work-for-the-sustainable-development-goals/the-imperative-of-blended-finance_9789264288768-6-en. Cited 2019 Feb 20

[4] UNDP (2019). Business and the SDGs.

[5] United Nations Department of Economics and Social Affairs (2015). Addis Ababa Action Agenda: Third International Conference on Financing for Development.

[6] Kuruvilla S, Hinton R, Boerma T, Bunney R, Casamitjana N, Cortez R (2018). Business not as usual: how multisectoral collaboration can promote transformative change for health and sustainable development. BMJ.

[7] Schmidt-Traub G (2015). Investment Needs to Achieve the Sustainable Development Goals.

[8] Organization WH, Health FM of SA and. Health in all policies: Helsinki statement. Framework for country action: World Health Organization; 2014. Available from: https://apps.who.int/iris/handle/10665/112636. Cited 2019 Feb 28

[9] Remme M, Vassall A, Lutz B, Luna J, Watts C (2014). Financing structural interventions: going beyond HIV-only value for money assessments. AIDS..

[10] Nunes AR, Lee K, O’Riordan T. The importance of an integrating framework for achieving the Sustainable Development Goals: the example of health and well-being. BMJ Glob Health. 2016:1 Available from: https://www.ncbi.nlm.nih.gov/pmc/articles/PMC5321355/. Cited 2019 Mar 27. 10.1136/bmjgh-2016-000068 PMC5321355 28588955

[11] Kuruvilla S, Schweitzer J, Bishai D, Chowdhury S, Caramani D, Frost L (2014). Success factors for reducing maternal and child mortality. Bull World Health Organ.

[12] Hardee K, Gay J, Croce-Galis M, Peltz A. Strengthening the enabling environment for women and girls: what is the evidence in social and structural approaches in the HIV response? J Int AIDS Soc. 2014:17 Available from: https://www.ncbi.nlm.nih.gov/pmc/articles/PMC3887370/. Cited 2018 Nov 19. 10.7448/IAS.17.1.18619 PMC3887370 24405664

[13] Remme M, Siapka M, Vassall A, Heise L, Jacobi J, Ahumada C, et al. The cost and cost-effectiveness of gender-responsive interventions for HIV: a systematic review. J Int AIDS Soc. 2014:17 Available from: https://www.ncbi.nlm.nih.gov/pmc/articles/PMC4221500/. Cited 2018 Sep 5. 10.7448/IAS.17.1.19228 PMC4221500 25373519

[14] Remme M, Martinez-Alvarez M, Vassall A (2017). Cost-effectiveness thresholds in Global Health: taking a Multisectoral perspective. Value Health.

[15] Robinson LA, Hammitt JK, Jamison DT, Walker DG (2019). Conducting benefit-cost analysis in low- and middle-income countries: introduction to the special issue. J Benefit-Cost Anal.

[16] Wilkinson T, Sculpher MJ, Claxton K, Revill P, Briggs A, Cairns JA (2016). The international decision support initiative reference case for economic evaluation: an aid to thought. Value Health.

[17] World Health Organization. Adelaide Statement on Health in All Policies: moving towards a shared governance for health and well-being: report from the International Meeting on Health in All Policies, Adelaide 2010. Geneva; Adelaide, S. Aust.: World Health Organization ; Government of South Australia; 2010. 10.1093/heapro/daq034 20484541

[18] World Health Organization. The world health report 2000 - Health systems: improving performance. Geneva; 2000. Available from: https://www.who.int/whr/2000/en/

[19] McDaid D, Park A-L (2016). Evidence on financing and budgeting mechanisms to support intersectoral actions between health, education, social welfare and labour sectors.

[20] Mason A, Goddard M, Weatherly H, Chalkley M (2015). Integrating funds for health and social care: an evidence review. J Health Serv Res Policy.

[21] Corbin JH, Jones J, Barry MM (2018). What makes intersectoral partnerships for health promotion work? A review of the international literature. Health Promot Int.

[22] World Health Organization. Chapter 1: Where are we now? World Health Report: Health Systems FInancing The Path to Universal Health Coverage. Geneva; 2010. Available from: https://www.who.int/whr/2010/10_chap01_en.pdf?ua=1. Cited 2019 Mar 27

[23] UNDP (2019). Financing across sectors for sustainable development: Guidanc Note.

[24] Ham C, Heenan DA, Longley M, Steel DR (2013). Integrated care in Northern Ireland, Scotland, and Wales: lessons for England.

[25] Scotland NHS (2011). Reshaping Care for Older People - a Programme for change 2011–2021.

[26] Harris J, Nguyen P, To Q, Hajeebhoy N, Phan L, Vu H, et al. Improvement in provincial plans for nutrition through targeted technical assistance and local advocacy in Vietnam. FASEB J Conf. 2015;29.

[27] Team JI (2015). Reshaping Care for Older People Change Fund: building on Progress.

[28] Audit Scotland. Reshaping care for older people Impact report. 2014. https://www.audit-scotland.gov.uk/report/reshaping-care-for-older-people-impact-report.

[29] Curry N, Ham C (2010). Clinical and service integration the route to improved outcomes.

[30] Kodner DL (2002). The quest for integrated systems of care for frail older persons. Aging Clin Exp Res.

[31] Béland F, Hollander MJ (2011). Integrated models of care delivery for the frail elderly: international perspectives. Gac Sanit.

[32] Heymann J, Hertzman C, Barer M, Evans R (2006). Healthier societies: from analysis to action.

[33] Clary A, Riley T. Pooling and braiding funds for Health-related social needs: lessons from Virginia’s Children’s services act: National Academy for State Health Policy; 2016.

[34] Eyles J, Brimacombe M, Chaulk P, Stoddart G, Pranger T, Moase O. What determines health? To where should we shift resources? Attitudes towards the determinants of health among multiple stakeholder groups in Prince Edward Island, Canada. Soc Sci. 2001;9. 10.1016/s0277-9536(00)00445-7 11762887

[35] Robison W, Chelala C, Freund P, Graybill E (2004). A healthy child in a healthy school environment - a look at CHANGES: program in Zambia.

[36] Freund P, Graybill E, Keith N (2005). Health and Education working together - a case study of a successful school Health and nutrition model.

[37] Education MO (2006). National School Health and Nutrition Policy.

[38] Okemo M, Sharif S. Kenya National School-Based Deworming Programme Year 1 (2012–2013) Results. Ministry of Education, Science and Technology and Ministry of Health; 2012.

[39] Rotich L, Maina W. Kenya National School-Based Deworming Programme Year 2 Report (April 2013–March 2014). Kenya: Ministry of Education, Science and Technology and Ministry of Health; 2013. https://ciff.org/documents/16/Kenya_National_SchoolBased_Deworming_Programme_Year2_evaluation.pdf.

[40] Rotich L, Kioko J. Kenya National School-Based Deworming Programme year 3 (2014–2015) results: Ministry of Education, Science and Technology and Ministry of Health; 2014. Available from: https://static1.squarespace.com/static/546f9316e4b0ced8102e4c74/t/583c5950414fb50504d8a8b4/1480350036170/NSBDP+Y3+Results+Booklet.pdf

[41] Mwandawiro CS, Nikolay B, Kihara JH, Ozier O, Mukoko DA, Mwanje MT (2013). Monitoring and evaluating the impact of national school-based deworming in Kenya: study design and baseline results. Parasit Vectors.

[42] O’Brien M, Bachmann MO, Jones NR, Reading R, Thoburn J, Husbands C (2009). Do integrated children’s services improve children’s outcomes?: evidence from england’s children’s trust pathfinders. Child Soc.

[43] Humphries R, Wenzel L (2015). Options for integrated commissioning.

[44] Stokes J, Lau Y-S, Kristensen SR, Sutton M (2017). Does pooling health & social care budgets improve quality and lower costs?.

[45] Better Care Fund: policy framework. :13.

[46] National Audit Office. Health and social care integration. 2017. https://www.nao.org.uk/wp-content/uploads/2017/02/Health-and-social-care-integration.pdf.

[47] Audit Commission for Local Authorities (2009). Means to an end: joint financing across health and social care: health national report.

[48] Social Care, Ageing and disability (2017). Integration and better Care fund policy framework 2017 to 2019.

[49] Humphries R, Wenzel L. Options for integrated commissioning. :64.

[50] Allebeck P (2008). Mapping household-based Health security - the case of Sweden. Soc Theory Health.

[51] Wihlman U, Lundborg CS, Axelsson R, Holmström I. Barriers of inter-organisational integration in vocational rehabilitation. Int J Integr Care. 2008:8. Cited 2019 Mar 15. Available from. 10.5334/ijic.234/. 10.5334/ijic.234 PMC2497404 18690291

[52] Alexanderson K, Norlund A (2004). Swedish council on technology assessment in Health Care (SBU). Chapter 1. Aim, background, key concepts, regulations, and current statistics. Scand J Public Health Suppl.

[53] Segal L, Dunt D, Day SE (2004). Introducing coordinated care (2): evaluation of design features and implementation processes implications for a preferred health system reform model. Health Policy.

[54] Gardner K, Sibthorpe B (2002). Impediments to change in an Australian trial of coordinated care. J Health Serv Res Policy.

[55] Battersby MW (2005). Health reform through coordinated care: SA HealthPlus. BMJ..

[56] Bergman H, Béland F, Lebel P, Contandriopoulos A-P, Tousignant P, Brunelle Y (1997). Care for Canada’s frail elderly population: fragmentation or integration. Can Med Assoc J.

[57] Scott R, Boyd R. Interagency performance targets a case study of New Zealand’s results Programme: IBM Centre for The Business of Government; 2017.

[58] Scott AM, Li J, Oyewole-Eletu S, Nguyen HQ, Gass B, Hirschman KB (2017). Understanding facilitators and barriers to Care transitions: insights from project ACHIEVE site visits. Jt Comm J Qual Patient Saf.

[59] Treasury T (2015). Cross-agency funding framework guidance for funding cross-agency initiatives.

[60] Batley R, Cabral L, Souza C (2007). Sector Wide Approaches in Brazil: Features, drivers and emerging lessons.

[61] Persaud Amlata (2017). Integrated planning for education and development. European Journal of Education.

[62] Burdescu R, Parel C. Brazil: Designing a New Loan Prototype to Meet Client Needs: World Bank; 2006.

[63] Unit F a. International bank for reconstruction and development program appraisal document on a proposed loan. The amount of us$350 million to the state of ceará, brazil with the guarantee of the federative republic of brazil to strengthen service delivery: FOR G. World Bank; 2013.

[64] <IMPLEMENTATION COMPLETION AND RESULTS REPORT (IBRD-73210) ON A LOAN IN THE AMOUNT OF US$ 149.750 MILLION TO THE STATE OF CEARÁ, BRAZIL FOR A CEARÁ MULTI-SECTOR INCLUSION DEVELOPMENT PROJECT.pdf>.

[65] Road Safety Partnership Grant, 2007–09 Schemes: Headline Impact Report. :20.

[66] King B, Surtees-Goodall S, Jeanes M. The Road Safety Partnership Grant Programme Summary Report of Impact of Round Two Projects and Progress; 2011. https://assets.publishing.service.gov.uk/government/uploads/system/uploads/attachment_data/file/4004/report.pdf.

[67] Transport D f. Review of the road safety partnership. Grant scheme: Department for Transport; 2009.

[68] Hultberg E-L, Glendinning C, Allebeck P, Lonnroth K (2005). Using pooled budgets to integrate health and welfare services: a comparison of experiments in England and Sweden. Health Soc Care Commun.

[69] Löfström M (2010). Inter-organizational collaboration projects in the public sector: a balance between integration and demarcation. Int J Health Plann Manag.

[70] Insurance C o (2014). Finsam – a follow-up of nancial coordination of rehabilitation measures.

[71] Ball R, Forbes T, Parris M, Forsyth L (2010). The evaluation of partnership working in the delivery of Health and social Care. Public Policy Adm.

[72] Board GG (2010). Community Health And Care Partnerships With Glasgow City Council.

[73] Welle K, Tucker J, Nicol A, Evans B (2009). Is the water sector lagging behind education and health on aid effectiveness? Lessons from Bangladesh, Ethiopia and Uganda. Water Altern.

[74] Management OP. A joint evaluation Uganda’s plan for the modernisation of agriculture: Ministry of Foreign Affairs Denmark; 2005.

[75] Gandhi G (2015). Charting the evolution of approaches employed by the global Alliance for vaccines and immunizations (GAVI) to address inequities in access to immunization: a systematic qualitative review of GAVI policies, strategies and resource allocation mechanisms through an equity lens (1999-2014). BMC Public Health.

[76] A Joint Evaluation of Uganda’s Plan for the Modernisation of Agriculture. Denmark: Ministry of Foreign Affairs Denmark; 2005. Available from: http://www.netpublikationer.dk/um/6207/pdf/200506PMAevaluation.pdf

[77] MSPAS MO. n. d. Intersectorial experience in the empowerment of adolescent girls republic of el. Salvador, Central: America;

[78] Hainsworth G, Zilhao I. From inception to large scale: the Geração Biz Programme in Mozambique, vol. 44: WHO; 2009.

[79] Osman NB, Zilhao I (2009). Sexual and reproductive rights of young people: understanding and meeting the need. Increasing access and demand to sexual and reproductive health and rights among adolescents in Mozambique. Int J Gynecol Obstet.

[80] Berry C, Kaplan SA, Reid A, Albert S (2009). The viability of community partnerships initiated by external funders. Public Health Rep.

[81] Chandra-Mouli V, Gibbs S, Badiani R, Quinhas F, Svanemyr J. Programa Geração Biz, Mozambique: how did this adolescent health initiative grow from a pilot to a national programme, and what did it achieve? Reprod Health. 2015:12. Cited 2019 Mar 15. Available from. 10.1186/1742-4755-12-12. 10.1186/1742-4755-12-12 PMC4429477 25971669

[82] Curry N, Harris M, Gunn L, Pappas Y, Blunt I, Soljak M, et al. Integrated care pilot in north west London: a mixed methods evaluation. Int J Integr Care. 2013:13. Cited 2019 Mar 15. Available from. 10.5334/ijic.1149/. 10.5334/ijic.1149 PMC3807631 24167455

[83] Pike B, Mongan D. The integration of health and social care services. :137.

[84] Miller R, Dickinson H, Glasby J (2011). The vanguard of integration or a lost tribe? Care trusts ten years on. Health.

[85] Evans D, Forbes T (2009). Partnerships in Health and social Care: England and Scotland compared. Public Policy Adm.

[86] Wistow G, Waddington E (2006). Learning from doing: implications of the barking and Dagenham experience for integrating Health and social Care. J Integr Care.

[87] Sillett J (2008). Clarifying joint financing arrangements - a briefing paper for health bodies and local authorities. Audit commission.

[88] National Audit Office UK (2012). Healthcare across the UK- a comparison of the NHS in England, Scotland, Wales and Northern Ireland.

[89] Heenan D, Birrell D (2009). Organisational integration in Health and social Care: some reflections on the Northern Ireland experience. J Integr Care.

[90] Alper J, Thompson D, Baciu A. Exploring Opportunities for Collaboration Between: National Academy of Sciences; 2015.

[91] Flynn M, Hayes C. Blending and Braiding Funds To Support Early Care and Education Initiatives. New York: The Finance Project; 2003.

[92] Learning MD (2011). Policy brief: increasing early childhood programs through blended and braided funding.

[93] Early Care and Child Consortium (2013). New Mexico policy facts - blending and braiding funding to support high quality child Care.

[94] Hudson B (1999). Joint commissioning across the primary health care-social care boundary: can it work?. Health Soc Care Commun.

[95] Soljak M, Cecil E, Gunn L, Broddle A, Hamilton S, Tahir A (2013). Quality of care and health outcomes.

[96] Steeden A (2013). The integrated Care pilot in north West London. London J Prim Care.

[97] Bardsley M, Smith J, Car J (2013). Evaluation of the first year of the inner north West London integrated Care pilot.

[98] Greaves F, Pappas Y, Bardsley M, Harris M, Curry N, Holder H, et al. Evaluation of complex integrated care programmes: the approach in North West London. Int J Integr Care. 2013:13. Cited 2019 Mar 15. Available from. 10.5334/ijic.974/. 10.5334/ijic.974 PMC3653284 23687478

[99] <Transforming the NHS in North West London Integrating health and social care with the leadership of local GPs and working in partnership with NHS England.pdf>.

[100] NHS Department of Health (2001). Guide to integrating community equipment services.

[101] Health Authorities, England (2003). The community Care (delayed discharges etc.) act 2003- guidance for implementation.

[102] Roll J, Wright K (2002). The community Care (delayed discharges etc) bill.

[103] Henwood M (2006). Effective partnership working: a case study of hospital discharge. Health Soc Care Commun.

[104] Lewis R, Glasby J (2006). Delayed discharge from mental health hospitals: results of an English postal survey. Health Soc Care Commun.

[105] Wanless D. Securing our Future Health: Taking a Long-Term View. 2002:179. https://www.yearofcare.co.uk/sites/default/files/images/Wanless.pdf.

[106] Rantala R, Larsen M, Gulis G, Koudenburg O, Armada F. Intersectoral action on health in urban settings – the experience of Varde Municipality, Denmark, 2007–201. : 1.

[107] Garg R, Karpati A, Leighton J, Perrin M, Shah M (2003). Asthma facts: second edition.

[108] Musuva RM, Matey E, Masaku J, Odhiambo G, Mwende F, Thuita I, et al. Lessons from implementing mass drug administration for soil transmitted helminths among pre-school aged children during school based deworming program at the Kenyan coast. BMC Public Health. 2017:17. Cited 2019 Mar 15. Available from. 10.1186/s12889-017-4481-7. 10.1186/s12889-017-4481-7 PMC5471907 28615011

[109] Suhonen M, Paasivaara L. Project work in Finnish KASTE projects. :17.

[110] Larsen M, Rantala R, Koudenburg OA, Gulis G (2014). Intersectoral action for health: the experience of a Danish municipality. Scand J Public Health.

[111] Vuorenkoski L (2008). New national development programme. National Institute for Health and Welfare THL.

[112] Kallinen S. National Development Plan for Social Welfare and Healthcare (Kaste Programme) 2012-2015 Final report: Ministry of Social Affairs and Health; 2016.

[113] Health M o. The National Development Programme for Social Welfare and Health: Care THE KASTE PROGRAMME; 2012. p. 2012.

[114] Kehittämis K, Kaste O. Sosiaali- ja terveydenhuollon. :140.

[115] Taylor M. Bristol Impact Fund: coordinating council grant streams - case study: Institute for Voluntary Action Research; 2017. Available from: https://www.ivar.org.uk/publication/bristol-impact-fund-coordinating-council-grant-streams/

[116] Sharma B, Nam E (2017). A healthy City project: a case study of Wonju City, South Korea and its relevance to the cities in Nepal. J Gandaki Med Coll Nepal.

[117] Stoddart GL, Eyles JD, Lavis JN, Chaulk PC. Reallocating Resources across Public Sectors to Improve Population Health: Oxford University Press; 2006. Cited 2019 Mar 27. Available from. 10.1093/acprof:oso/9780195179200.001.0001/acprof-9780195179200-chapter-13.

[118] Glasby J, Peck E (2004). Care trusts: partnership working in action.

[119] Hendry A. Creating an Enabling Political Environment for Health and Social Care Integration. Int J Integr Care. 2016:16. Cited 2019 Mar 15. Available from. 10.5334/ijic.2531/. 10.5334/ijic.2531 PMC5354203 28316547

[120] Government TS. National Health and wellbeing outcomes: a framework for improving the planning and delivery of integrated health and social care services: The Scottish Government; 2015.

[121] Godden S, McCoy D, Pollock A (2009). Policy on the rebound: trends and causes of delayed discharges in the NHS. J R Soc Med.

[122] WHO Regional Office for Europe (2018). Multisectoral and intersectoral action for improved health and well-being for all: mapping of the WHO European Region Governance for a sustainable future: improving health and well-being for all.

[123] Chircop A, Bassett R, Taylor E (2015). Evidence on how to practice intersectoral collaboration for health equity: a scoping review. Crit Public Health.

[124] Mainali B, Luukkanen J, Silveira S, Kaivo-oja J (2018). Evaluating synergies and trade-offs among sustainable development goals (SDGs): explorative analyses of development paths in South Asia and sub-Saharan Africa. Sustainability.

[125] Nilsson M, Griggs D, Visbeck M (2016). Policy: map the interactions between sustainable development goals. Nat News.

[126] Word Health Organization (2018). Towards a Global Action Plan for Healthy Lives and Well-being for all.

[127] The South African AIDS COuncil. South Africa’s National Strategic Plan for HIV, TB and Malaria 2017–2022: SANAC; 2017. Available from: https://sanac.org.za/the-national-strategic-plan/. Cited 2019 Apr 16

[128] Buse K, Hawkes S (2015). Health in the sustainable development goals: ready for a paradigm shift?. Glob Health.

