# Supplementary material for: Financing intersectoral action for health: a systematic review of co-financing models
Source: Global Health. 2019 Dec 18;15:86. doi: 10.1186/s12992-019-0513-7 (PMC6918645; doi:10.1186/s12992-019-0513-7)
Supplement: Supplementary file 1 — Additional file 1. Case Descriptions, including country, financing mechanisms, outcomes data [file 12992_2019_513_MOESM1_ESM.docx]

**Additional Data**

**Table1: Integrative Model**

| **No** | **Case** | **Country** | **Co-financed intervention** | **Financial mechanism** | **Payers involved** | **Target population, level and scale of implementation** | **Legal/ regulatory framework** | **Evaluation** |
| --- | --- | --- | --- | --- | --- | --- | --- | --- |
| **1** | **Better Care Fund**  [1–6] | England | Mechanism for joint health & social care planning & commission | National level pooled budget with grant applications | Health – Department of Health  Social Care – Department for Communities and Local Government | National population | Health and Social Care Act (2012); Care Act (2014) | **Evaluation Methodology**: Quantitative (difference-in-difference, trend analysis); Qualitative (interviews)  **Output/Outcome affect:**  *Health*   - No significant mortality effects or service utilisation effects - Increase of 87,000 emergency hospital admissions between 2014/15-2015/16, against planned reduction of 106,000   *Social Care*   - Increase of 185,000 in delayed transfers of care between 2014/15-2015/16, against planned reduction of 293,000 - 628 permanent admissions of older people (age 65+) to residential and nursing care homes per 100,000 population in 2015/16, exceeding target of 659 per 100,000 - 82.7% of older people who were still at home 91 days after discharge from hospital receiving rehabilitation services in 2015/16, exceeding the target of 81.9%   *All*   - 90% of local areas agreed or strongly agreed that the delivery of the BCF plans had a positive impact on local integration   **Input/Process affect:**   - Original allocation of £3.8bn in 2015/16. £1.9bn coming from NHS allocations to CCGs, £1.1bn pre-existing transfer from NHS to social care. £0.8 from other health & care funding streams (e.g. Disabled Facilities Grant) - Ultimately £5.3bn was pooled in 2015/16 but still represents less than 5% of health & social care spending - 75% of expenditure went on social care (43%) and community care (32%) in 2015/16 |
| **2** | **Section 31 Health Act /National Health** **Service Act section 75**  [4,7]; | England | Mechanism for joint health & social care planning & commissioning | Pooled budget | Health – Clinical Commissioning Groups (CCGs)  Social Care – Local Authorities | National population | Health Act (1999);  NHS Act (2006) | **Evaluation Methodology**: Quantitative (trend)  **Output/Outcome affect:**  *Health*   - Reduction in teenage conception rates and obesity rates (Swindon) - Number of common assessments doubled in the first six months of 2009/10 (Swindon)   *Education*   - Improved educational attainment at Key Stage 4 (Swindon)   **Input/Process affect:**   - Swindon established section 75 agreement in 2008. In 2015 £136m is dedicated (£47m from CCG & £89m from Local Authority). Estimated set up cost of £10,000. - In 2006 Oxfordshire & Buckinghamshire NHS Trust entered agreement with 2 Local Authorities for MH services with pool of £38.6m |
| **3** | **Greater Manchester Combined Authority**  [5,8,9]; | England | Mechanism for joint health & social care planning & commissioning as part of The Greater Manchester (GM) Health and Social Care Devolution whereby full local responsibility is assumed for NHS funding streams | Unclear | Health  Social Care | Local population | Local Government Act (2000); Local Democracy, Economic Development & Construction Act (2009); Association of Greater Manchester Authorities MOU (2015); The Cities and Local Government Devolution Act (2016) | **Evaluation Methodology**: No evaluation found  **Output/Outcome affect:**  **Input/Process affect:**   - Total health & social care budgets worth approximately £6 billion in 2015/16 - Joint commissioning board (JCB) established, comprising local authorities, CCGs and NHS England |
| **4** | **Integrated health & social care commissioning**  [5,10–12] | Scotland | Mechanism for joint health & social care planning & commissioning | Pooled budget (with lead agency or integrated joint board) | Health – Health Boards  Social Care – Local Authorities | National population | Public Bodies (Joint Working) Act (2014) | **Evaluation Methodology:** Quantitative (trend)  **Output/Outcome affect**: No documentation found  **Input/Process affect:** No documentation found |
| **5** | **Reshaping Care for Older People programme**  [13–17] | Scotland | Programmes implementing holistic care & interventions which benefit elderly individuals and carers | National level pooled budget with grant applications (joint strategic commissioning plans) | Health  Social Care  Housing | Elderly |  | **Evaluation Methodology**: Qualitative (partnership self-assessments of initiatives)  **Output/Outcome affect:**  *Health*   - 6Rate of emergency bed days for people 75+ has fallen by 10.2% between 2009/10 & 2013/14 (target is 12% reduction by 2015)   *Social Care*   - 17% fewer older people taken to hospital after a minor fall - 2 million more days in own home than ‘expected’   **Input/Process affect:**   - 1,456 new houses for older & disabled individuals constructed between 2011-2013 - £300M Change Fund distributed to Local Partnerships between 2011-15 - Increase in care at home spending for 65+ from 6.7% of total health & care expenditure in 2009/10 to 9% in 2010/11 - 39% Change Fund provided support for carers - Change Fund expenditure on initiative in hospital or care homes has fallen from 25% (2011/12) to 10% (2014/15), mirrored by increased expenditure on preventative & anticipatory care |
| **6** | **Care Trusts**  [18][19] [20]  [21]; [22]; [10]; [23–27] | England | Mechanism for joint health & social care planning, commissioning & provision | Full organisational & budgetary integration | Health – Clinical Commissioning Groups (CCGs)  Social Care – Local Authorities | National population | NHS Plan (2000); Health Act (1999); NHS Act (2006) | **Evaluation Methodology**: Qualitative (national survey, workshops, semi-structured interviews); Quantitative (trend)  **Output/Outcome affect:**  *Health*   - No evidence of improved health outcomes - No evidence of greater efficiency with trends similar to PCTs - Variations in the success of initiatives (Torbay Care Trust perceived as a success but other cases less positive)   **Input/Process affect:**   - Resource redistribution went towards previously unmet need rather than improving existing care - Positive perception of integration of health & social care professionals |
| **7** | **Cumbria PCT (Integrated care pilot)**  **(2009-2011)**  [28]  [29];  [30] | England | Case management of patients with high risk of admission through bringing together GP & community services at 3 project sites | Pooled budget (budgets devolved from the PCT to each project sites to enable joint commissioning) | Health  Social Care | Whole local population covered but individuals at risk of admission targeted | Dept. of Health Programme of Integrated Care Pilots (2008) | **Evaluation Methodology:** Quantitative (difference-in-difference); Qualitative (questionnaires)  **Output/Outcome affect:**  *Health*   - 9% increase in non-elective admissions (for 6 ICP sites focusing on case management combined incl. Cumbria) - Fall in elective admissions & outpatient attendance by 21% & 22% respectively (for 6 ICP sites focusing on case management combined incl. Cumbria) - Reduction of in/outpatient costs of 9% in six months following intervention - Outpatient attendance reduced by 5% two years after intervention   I**nput/Process affect:**   - PCT given £180,000 to cover start-up costs & evaluation |
| **8** | **Darlington Community Care Project**  **(1989)**  [31] | England | Provision of alternative carte at home by multi-purpose carers deployed by case managers for patients in long-term hospital care | Pooled budget (Service managers held up to 20 devolved individual capitation budgets) | Health  Social Care | Frail elderly patients requiring long-term care |  | **Evaluation Methodology:** Quantitative (matching); Qualitative (interviews)  **Output/Outcome affect:**  *Health*   - Increase in patient morale for intervention group   *Social Care*   - No evidence of greater stress on carers   **Input/Process affect:**   - Service managers individual capitation budgets equivalent to 67% of the cost of institutional care - Total cost of care lower for intervention group largely due to lower institutional care utilisation |
| **9** | **Hertfordshire Integrated specialist mental health service**  **(2001)**  [32] | England | Provision of specialist, integrated mental health, learning disability & drug & alcohol services by Hertfordshire Partnership Trust | Pooled budget (joint commissioning through integrated joint board) | Health – Primary Care Trusts  Social Care – Local Authorities | Adults and children with mental health problems, learning disability or using drug and alcohol services | Section 31 of the Health Act (1999) | **Evaluation Methodology:** Qualitative (focus groups, interviews, postal survey)  **Output/Outcome affect:**  *Health*   - Users reported a perception of benefits stemming from the integration   *Social Care*   - Carers also reported positive perception of integration and increased responsiveness to carer concerns   **Input/Process affect:**   - Total pooled budget was £160m in 2002/3 - The anticipated reduced pressure on Community Mental Health Teams did not materialise - Self-reported benefit of co-location of multi-disciplinary teams - Self-reported scores of perceived team effectiveness fell between 2002-2004 |
| **10** | **North West London Integrated Care Pilot**  **(2011)**  [22,33,34];  [35]; [36];  [37]; [38] | England | Proactive care planning across care settings by doctor-led multi-disciplinary teams, targeted at high-risk individuals with complex needs; care delivered by community teams; use of risk stratification tool (CPM: Combined Predictive Model), and data sharing systems; aligned incentive structure | Joint commissioning (lead commissioning)  Aligned financial incentives (organisations must agree to share savings to join pilot) | - Health - Social Care | Individuals with diabetes & individuals aged 75+ |  | **Evaluation Methodology**: Quantitative (matching); Qualitative (focus groups, interviews, participant observation, surveys)  **Output/Outcome affect:**  **Input/Process affect**:   - Integrated Management Board (IMB) was established - Pilot received £10M from the London Strategic Health Authority (National Health Service London), which enabled investment in governance arrangements, a support team and a data-sharing platform |
| **11** | **Oxfordshire pooled budgets/lead commissioning**  [39] | England | Unclear | Pooled budget | Health  Social Care |  |  | **Evaluation Methodology:** No evaluation found  **Output/Outcome affect:**  **Input/Process affect:** |
| **12** | **Programs of All-Inclusive Care for the Elderly (PACE)**  [40,41]; | United States | Day health centre based-programme delivering a comprehensive range of health & social care to postpone/avoid institutionalisation of enrolled older people | Pooled budget (pooled capitation of Medicaid & Medicare payments) | Health – Medicare  Health – Medicaid  Social Care | Elderly (55+) nursing home-certifiable | The Balanced Budget Act (1997) | **Evaluation Methodology:** Quantitative (matching)  **Output/Outcome affect:**  *Social Care*   - PACE enrolees had 50% less hospital use than control group - Admission to nursing home 20% lower than control with 16 fewer bed days if admitted   *Health*   - 43% PACE-enrolees self-reporting good health compared to 37% non-enrolees - Estimates of 5-15% cost saving compared to standard fee for service care - Higher ambulatory care usage (93% compared to 74% for control group)   **Input/Process affect:**   - 107 PACE programmes operating in 2015, largest programme has 2,500 enrolees - Programme assumes financial risk for population giving it flexibility to provide needed services |
| **13** | **Be-Life Programme (Better life for the most ill elderly people program)**  [42];  [43]; [44] | Sweden | National programme for improving quality of health & social care for older people | Incentive grants with requests for (joint) proposals | Health – Ministry of Health and Social Affairs  Social Care – Swedish Association of Local Authorities and Regions (SALAR) | Elderly | Annual framework agreements between SALAR & Swedish Government | **Evaluation Methodology**: Quantitative (trend); Qualitative (interviews)  **Output/Outcome affect:**  *Social Care*   - Most regions managed small reductions in avoidable inpatient care & readmission within 30 days   *Health*   - All regions reduced use of specified medications   **Input/Process affect:**   - Conclusion that coordination between health & social care hasn’t increased - Increase in number of registered risk assessments on Senior Alert Registry |
| **14** | **Children’s Trust Pathfinders**  [45] | England  (2003-06) | Local cross-sector partnerships promoting greater integration of professionals providing children’s services. Pathfinders established inter-agency governance arrangements to better coordinate planning, commissioning & delivery with a number of statutory obligations | National level pooled budget with grant applications | Health – Department of Health  Education – Department of Education and Skills Development | Children | Children Act (2004) | **Evaluation Methodology:** Quantitative (trend); Qualitative (document review, surveys, informant interviews)  **Output/Outcome affect:**  *Health*   - No evidence of better outcomes for more integrated areas - Pathfinders focusing on ‘all children’ rather than defined groups showed more progress - 25/35 survey respondents gave local specific examples of arrangements improving outcomes   **Input/Process affect:**   - 75/100 Local Authorities bid for funding - 35 Pathfinders received funding between £60,000-£100,000 per annum - Coverage of 20% of England’s children - Local Authorities established Child Services Authorities - 100% Pathfinders had a cross-sector governance structure such as a Children’s Trust board - 43% had joint delivery arrangements such as multi-agency teams - 66% of Pathfinders moved towards pooling budgets in at least some service areas by 2006 |
| **15** | **FINSAM**  [46]; [47]; [48,49] | Sweden (1993-present) | Enabled the allocation of up to 10% of the social insurance’s resources for sickness & rehabilitation funds to be used for health services through local agreements on shared initiatives | Aligned budgets | Health – County Councils  Social Care – National Social Insurance Board | Individuals in need of rehabilitation services | Act on Financial Coordination of Rehabilitation Measures (2004) made trial permanent | **Evaluation Methodology:** Quantitative (trend)  **Output/Outcome affect:**  *Health*   - Reduction in the ‘unhealth’/’incapacity rate’ (combined measure of transfer costs due to sickness absence/early retirement) of 2.5% in trial areas compared to a 2.1% increase across country between 1993-95 - Public expenses reduced in 4 of 5 trial areas   *Education/Employment*   - Number of participants in employment or education increased by 20% in 2014 - Proportion of participants without income support increased by 12-14% in 2014   **Input/Process affect:**   - FINSAM allocated SEK 560 million per annum between 2012-15 - Only 1-2% instead of 10% allowed ever reallocated |
| **16** | **FRINSAM**  [50,51] | Sweden |  |  | Health – County Councils  Social Care – Social Welfare Services  Social Insurance – National Social Insurance Board |  |  | **Evaluation Methodology**: No evaluation found  **Output/Outcome affect:**  **Input/Process affect:** |
| **17** | **SOCSAM**  **(1994-present)**  [50–52]; | Sweden | Enabled the creation of a joint SOCSAM budget with the health, social care & social insurance sectors contributing up to 5% of their local budgets. Local joint governance boards are free to dictate the use of the funds | Local pooled budgets with joint governance boards | Health – Ministry of Health and Social Affairs  Social Insurance – National Social Insurance Board | Individuals suffering long-term illness affecting employment | SOCSAM Trial legislation (1994) | **Evaluation Methodology:** Quantitative (trend); Qualitative (interviews, questionnaires)  **Output/Outcome affect:**  *All*   - No observable reduction in costs   *Health*   - No observable improvement in patient health status under the co-financing arrangements compared to controls   **Input/Process affect:**   - Eight trial areas contributed differing proportions of their budgets - In smaller areas, the joint SOCSAM boards took over all 3 authorities usual activities including services for people of all ages - SOCSAM areas believed to have a positive effect on staff and organisation |
| **18** | **Social Services Modernization Fund**  **(1998)**  [26,33]; | England | Join plans by the health & social care sector for services that encourage independence through improvements in rehabilitative or preventive services | Pooled grant (budget) requiring cross-sector applications | Health – Department of Health; Clinical Commissioning Groups; NHS England  Social Care – The Social Services Committee | Individuals requiring care or at risk of losing their independence & carers | Modernising Social Services: White Paper (1998) | **Evaluation Methodology:** No evaluation identified  **Outcome affect:**  **Process affect**:   - Establishment of the fund worth £1,327M between 1999/2000-2001/02 |
| **19** | **The Home Loans Equipment Centre (HLEC)**  **(1980- )**  [53] [33]; [43] | England | Health & social care equipment to assist individuals living with disability or illness | Joint commissioning | Health  Social Care | Individuals requiring community or home equipment | Guide to Integrating Community Equipment (2001) | **Evaluation Methodology:** No evaluation identified  **Outcome affect:**  **Process affect:** |
| **20** | **Personal care at home project**  [33] | England | Personal Care Assistants providing home-based social & nursing support to individuals with high need | Joint Commissioning | Health  Social Care | Elderly |  | **Evaluation Methodology:** No evaluation identified  **Outcome affect:**  **Process affect:** |
| **21** | **Home for Good Funders Collaborative**  **(2011- )**  [54]  [55–58]; | United States | Provision of permanent supportive housing and services for chronically homeless individuals | Pooled budget with grant applications (for unified request for proposal application for multiple funding streams) | Health – LA County Department of Public Health; LA County Department of Mental Health Services  Social Care – Services Veterans Administration of Greater LA; City of Pasadena; City of Santa Monica; City of West Hollywood; LA Community Investment Department; LA County Fourth District Supervisor Don Knabe  Housing – Housing Authority of the City of LA; US Department of Housing and Urban Development | Homeless individuals |  | **Evaluation Methodology**: Quantitative (trend); Qualitative (Interviews, annual stakeholder surveys)  **Outcome affect:**  *Housing*   - 17,598 individuals housed between 2011-14   **Process affect:**   - Leveraged seed investment to raise US$562.1M committed to the initiative between 2011-15 - Single coordinated entry system established to screen, match and provide services prioritising the most vulnerable individuals - Identified increase in the involvement and support of local politicians and organisations |
| **22** | **Waitakere Abuse and Trauma Counselling Service**  **(1990)**  [59] | United States | Provision of counselling & therapy for victims of abuse, trauma & family violence | Unknown  Funded by various sources:  Ministry of Social Development contract and;  Fee for service funding through Accident Compensation Corporation (ACC) and the Ministry of Justice and;  Small voluntary client donations (often nothing)  Philanthrophic sector to cover shortfalls | Health – Accident Compensation Corporation (ACC)  Social Care – Ministry of Social Development  Justice – Ministry of Justice | Individuals who have suffered abuse |  | **Evaluation Methodology:** No evaluation identified  **Outcome affect:**  **Process affect:** |
| **23** | **Contra Costa County Community Services Dept. coordinated funds for early education**  **(National Early Care and Education Collaborative Initiative)**  **(1998)**  [60–63] | United States | Full-day full-year integrated early education & support services | Joint commissioning | Child care  Education  Social services | Children |  | **Evaluation Methodology:** No evaluation identified  **Outcome affect:**  **Process affect:** Merged previously separate Head Start & Child Development divisions into single Family & Children’s Services division within the Community Services Dept. |
| **24** | **Florida Partnership for School Readiness (National Early Care and Education Collaborative Initiative)**  **(1999**)  [60] | United States | County level School Readiness Coalition plans for early care & education in the community (incl. Even Start Literacy programs, Florida First Start, prekindergarten early intervention, subsidised child care, teen pregnancy programs etc.) | Pooled budget (State develops contract with coalitions which pools relevant funds for each program) | Child care – Department of Children & Families  Education – Department of Education  Health – Department of Health | Children | School Readiness Act (1999) | **Evaluation Methodology**: No evaluation identified  **Outcome affect:**  **Process affect**: |
| **25** | **Oregon’s Youth Transition Program**  **(1990- )**  [54,64]; | United States | Provision of extensive vocational rehabilitation services delivered by community-based transition specialists, vocational rehabilitation counsellors etc. to youth focusing on post-school planning, job training & placement | Pooled budget with grant applications (schools apply through a RFP with successful schools providing matching funds) | Education – Oregon Department of Education  Vocational rehabilitation – Oregon Vocational Rehabilitation Department  University – University of Oregon | Youth with disabilities |  | **Evaluation Methodology**: Quantitative (trend, matching); Qualitative (interviews)  **Outcome affect:**  *Education*   - Program participants have a 91% high school completion rate compared to 72% of students in special education during high school nationally   *Vocational rehabilitation*   - Average wage 12 months after program completion of US$9.64 higher than Oregon minimum wage (US$8.40 in 2009) - 77% of participants engaged in employment, post-secondary education or training 12 months after program completion   *All*   - Estimated cost per client is US$3,000   **Process affect:**   - Program has operated in 209 high-schools within Oregon since 1990 - 1,315 school & rehabilitation staff participated in training program between 2011-15 - Since inception the program has provided services to over 20,000 individuals |
| **26** | **Speech and Language Therapy in Enfield**  **(2011- )**  [65] | England | Speech & language therapy (SALT) | Joint commissioning | Health –Primary Care Trust  Education – Local Authority (Dedicated Schools Grant; Area Based Grant) | Children & youth | Children & Families Act (2014) | **Evaluation Methodology**: No evaluation identified  **Outcome affect:**  **Process affect:**   - Total annual spend of £1.96M on SALT in Enfield |
| **27** | **Speech and Language Therapy in Surrey**  **(2014)**  [66] | England | Speech & language therapy (SALT) | Joint commissioning | Health  Education | Children & youth | Children & Families Act (2014) | **Evaluation Methodology:** No evaluation identified  **Outcome affect:**  **Process affect:** |
| **28** | **Integrated Health & Social Services Board**  **(1973-present)**  [67]; [68]; [13] | Northern Ireland | Mechanism for joint health & social care planning, commissioning & provision | Full structural & budgetary integration | Health – Health and Social Care Boards  Social care– Health and Social Care Boards | National population | The Administrative Structure of Health and Personal Social Services in Northern Ireland (1969);  Health & Personal Social Services Order (1994) | **Evaluation Methodology:** Qualitative (interviews & focus groups); Quantitative (trend)  **Outcome affect**:  *Social Care*   - Reduced delays in hospital discharges   *All*   - No evidence of consistently higher measures of efficiency or effectiveness than England, Scotland or Wales   **Process affect:**   - Dept. of Health, Social Services & Public Safety has authority for both health & social services. - Services commissioned by 5 Health & Social Care Boards & provided by Health & Social Care Trusts - Integration achieved through division of health care into 9 programmes of care within each Trust to meet the complex needs of individuals - Individuals assigned named care workers - Health sector given priority in resource allocation over social care sector with 42% of funding going to acute care programme in 2009/10 - Health expenditure per capita 10.8% higher in NI than England while social care expenditure only 5.1% higher in 2010/11 - Integrated management structure where programme management roles are open across all professions - Performance targets almost entirely health related |
| **29** | **Australian Capital Territory (ACT) Coordinated Care trial - CCTR1**  **(1997-1999)**  [70]; [71]; [72]; [73]; [69] | Australia | Regional project whereby GP acted as care coordinator for participants developing comprehensive individualised care plans | Pooled budget (establishment of new purchasing organisation – Careplus – used capitation payments to providers) | Health – Medical Benefit Fund (MBS); Pharmaceutical Benefits Fund (PBS); Public Hospital Funding  Social care – ACT Community Care; Home and Community Care (HACC) | Individuals with complex or chronic illness | Council of Australian Governments reform agenda (1995) | **Evaluation Methodology:** Quantitative (RCT); Qualitative (interviews)  **Outcome affect:**  *Health*   - No significant difference in health outcomes between control & treatment group as measured by SF-36 - No significant difference in admission rates - Reduced length of stay for treatment group   **Process affect:**   - Program operated within existing resource envelope - No evidence that pooled budget led to more coordinated care or changes in service delivery |
| **30** | **The Illawarra Coordinated Care Trial - CCTR1**  **(1997-1999)**  [74]; [75]; [72]; [73]; [76] | Australia | Regional project whereby 16 care coordinators worked in collaboration with participants GPs to develop comprehensive individualised care plans | Pooled budget (establishment of new purchasing organisation – Care Net) | Health – The Health Insurance Commission; The Illawarra Area Health Service  Social care – The Home and Community Care program; The Department of Veterans’ Affairs | Individuals with complex illness (aged 65+) | Council of Australian Governments reform agenda (1995) | **Evaluation Methodology**: Quantitative (RCT)  **Outcome affect:**  *Health*   - No significant difference in health outcomes between control & treatment group as measured by SF-36 - Physical & social function of 35% & 45% of treatment group declined and remained constant respectively over the trial   *Social Care*   - Intervention group twice as likely to be admitted to residential care (7.5% vs. 3.3%)   **Process affect:**   - AUS$1.7M deficit at end of the trial (12.7% of allocated budget) |
| **31** | **North Eastern Health Care Network (Victoria) - CCTR1**  **(1997-1999)**  [72]; [73]; [77] | Australia | Regional project whereby GPs and separate care coordinators shared aspects of care coordination process | Unsure | Health – Medical Benefit Fund (MBS); Pharmaceutical Benefits Fund (PBS); Public Hospital Funding; Royal District Nursing Service  Social care – Home and Community Care programme; The Department of Veterans’ Affairs (DVA) | Individuals with complex illness | Council of Australian Governments reform agenda (1995) | **Evaluation Methodology:** Quantitative (RCT)  **Outcome affect:**  *Health*   - No significant difference in health outcomes between control & treatment group as measured by SF-36 - No significant difference in admission rate - No significant difference in length of stay   **Process affect:** |
| **32** | **Care 21 (South Australia) - CCTR1**  **(1997-1999)**  [72]; [73]; [77] | Australia | Regional project whereby 16 care coordinators worked in collaboration with participants GPs to develop comprehensive individualised care plans | Pooled budget | Health – Medical Benefit Fund (MBS); Pharmaceutical Benefits Fund (PBS); Public Hospital Funding (inpatient only); Royal District Nursing Service  Social care – Home and Community Care programme; The Department of Veterans’ Affairs (DVA) | Individuals with complex illness (aged 65+) | Council of Australian Governments reform agenda (1995) | **Evaluation Methodology**: Quantitative (geographic controls)  **Outcome affect:**  *Health*   - Significantly decrease in physical functioning of treatment group as measured by SF-36 - No significant difference in admission rate - Length of stay significantly longer in treatment group   **Process affect:** |
| **33** | **Hornsby Linked Care (New South Wales) - CCTR1**  **(1997-1999**)  [72]; [73]; [77] | Australia | Care coordinator (GP and non GPs) and GP (if not a care coordinator) and Super Care Coordinator (full-time non GPs). | Pooled budget | Health – Medical Benefit Fund (MBS); Pharmaceutical Benefits Fund (PBS); Public Hospital Funding; Royal District Nursing Service  Social care – Home and Community Care; Department of Veterans Affairs | Individuals with complex illness | Council of Australian Governments reform agenda (1995) | **Evaluation Methodology:** Quantitative (geographic controls)  **Outcome affect:**  *Health*   - No significant difference in health outcomes between treatment & control groups as measured by the SF-36   **Process affect:** |
| **34** | **CareWorks (Southern Region of Tasmania) - CCTR1**  **(1997-1999)**  [72]; [73]; [77] | Australia | Regional project whereby 16 care coordinators worked in collaboration with participants GPs to develop comprehensive individualised care plans | Unsure | Health – Medical Benefit Fund; Pharmaceutical Benefits Fund; Public Hospital Funding  Social care – Home and Community Care; Department of Veterans Affairs | Individuals with complex illness (aged 65+) | Council of Australian Governments reform agenda (1995) | **Evaluation Methodology:** Quantitative (geographic controls)  **Outcome affect:**  *Health*   - No significant difference in health outcomes between treatment & control groups as measured by the SF-36 - No significant difference in admission rate - No significant difference in length of stay   **Process affect:** |
| **35** | **Coordinated Health Care (CHC)  The CHC trial was linked to the CCTR1 North Eastern Health Care Network**  **(2003-2005)**  [76] | Australia | GP acted as care coordinator with service coordinator approach  (nurses). Home based health assessment, multidisciplinary care planning, service  coordination | Pooled budget | Health – Medical Benefit Fund; Pharmaceutical Benefit Fund  Social care – Home and Community Care | Individuals with complex illness | Council of Australian Governments reform agenda (1995) | **Evaluation Methodology:** Quantitative (RCT); Qualitative (focus groups)  **Outcome affect:**  *Health*   - No significant difference in general health or depression scores at baseline or after 6 months between treatment and control group - Control group had higher HrQoL at baseline but after 6 months this had fallen to same level as treatment group how experienced no change - Primary care utilisation remained constant for treatment group while inpatient utilisation decreased. Control group primary care utilisation fell but inpatient utilisation increased   **Process affect:** |
| **36** | **Team Care Health II (TCHII)  The TCHII trial was linked to the CCTR1 TeamCare Brisbane, Queensland**  **(2003-2005)**  [76] | Australia | GP acted as care coordinator with service coordinator approach (nurses). GP assessment, multidisciplinary care planning, service coordination | Pooled budget | Health – Medical Benefit Fund; Pharmaceutical Benefit Fund  Social care – Home and Community Care | Individuals with complex & chronic illness (aged 50+) | Council of Australian Governments reform agenda (1995) | **Evaluation Methodology:** Quantitative (RCT); Qualitative (focus groups)  **Outcome affect:**  *Health*   - Improved subjective well-being - Significant difference in general health scores, depression scores and HRQoL between treatment and control group after one year, with treatment group performing better in all measures - Treatment group had reduced inpatient utilisation & costs relative to control group   *Social Care*   - Substitution of inpatient care for community care for treatment group   *All*   - Total cost of service provision increased for both grouped but significantly more for control group - Belief that the trial would have eventually become cost neutral even accounting for cost of care coordination   **Process affect:**   - 2,720 participants enrolled in the trial – 1,774 intervention group participants and 946 control group participants - Average enrolment duration of 18 months |
| **37** | **Programme of Research to Integrate Services for the Maintenance of Autonomy (PRISMA)**  [78]; [79]; [74]; [80]; [81] [82] | Canada | Integrated service delivery network providing single entry point, case management, service coordination with comprehensive individualised care plans for patients developed between providers | Budgets negotiated between participating organisations through a joint governing board | Health  Social Care | Elderly (65+) with presence of significant functional disability |  | **Evaluation Methodology**: Quantitative (matching)  **Outcome affect:**  *Health*   - Smaller proportion of intervention group experienced ‘functional decline’ (death, institutionalisation, worse disability) compared to control group (31.3% vs. 49.1%) - Lower desire to be institutionalised in intervention group   *Social Care*   - Lower care giver burden in intervention group - Hospital acute care utilisation rate similar but lower risk of return within 10 days for intervention group - Higher risk of institutionalisation in control group   **Process affect:**   - Small process effect as implementation region already had integrated health & social care system |
| **38** | **System of Integrated Care for Older Persons (SIPA)**  [74];; [41,72,83]; [84] | Canada  (1999-2001) | Community-based care model responsible for comprehensive range of primary & secondary health care & social services for elderly | Pooled budget (supposed to be pooled capitation payment but in practise prepayment financing was not implemented and programmes just held pooled budget for services) | Health  Social Care | Elderly |  | **Evaluation Methodology**: Quantitative (RCT)  **Outcome affect:**  *Health*   - No differences in health outcomes or total costs between treatment & control group - Impact on costs was larger for individuals with several conditions indicating importance of targeting   **Process affect:**   - Multi-disciplinary team assumed total clinical control of participants - Physician participation was found to be challenging - SIPA not pursued as a permanent programme - SIPA found to be cost-neutral with reduction in institutional costs offset by higher community-care costs - Number of delayed discharges 50% lower in SIPA group compared to control - Decreased utilisation of all hospital-based services |
| **39** | **NHS Cross-charging**  **(2003-present)**  [85–88]; [89] | England | System of ‘reimbursement’ by social services to the relevant NHS organisation where discharge delays are caused solely by the failure of the social services authority to provide timely assessment or social care services | Cross charging (Mandatory daily penalties of £100-120 per day) | Health – relevant NHS organisations  Social Care – Local Authorities | Individuals in acute care | Community Care (Delayed Discharges etc.) Act 2003 | **Evaluation methodology:** Quantitative (trend)  **Outcome affect:**  *Social Care*   - Downward trend in delayed discharges prior to introduction of Delayed Discharges Act, however, trend has accelerated since implementation (64% reduction in delayed discharge from acute hospitals between 2001-2005)   *Health*   - Much more mixed picture regarding quality of patient’s experiences and targets on ‘bed blocking’ sometimes met at the expense of potential improvements in outcomes - Evidence of moving individuals directly into institutionalised care rather than efforts to return them to their own homes in order to facilitate discharge   **Process affect:**   - Delayed Discharge Grant (transferring £100 million from the NHS to local authorities for each full year of the operation of the reimbursement scheme) sought to address the problem of delayed discharge from a whole systems approach - Number of new duties and responsibilities established such as NHS bodies having a new statutory duty to notify social services of a patient’s likely need for community care services and a defined time scale (the ‘minimum interval’ of at least 3 days) for social services to complete the individual’s assessment and provide appropriate social care services - National Reimbursement Implementation team established to support local partnerships in strengthening discharge planning arrangements |
| **40** | **ADEL reform (National Reform of Elderly Care)**  **(1992-** | Sweden | Local authorities (responsible for social care) are required to pay county councils (who run hospitals) for care delivered to patients in hospital once a patient is deemed fully medically treated by a hospital doctor | Cross charging | **Health**  **Social Care** | Individuals in acute care |  | **Evaluation methodology:** No evaluation identified  **Outcome affect:**  **Process affect:**   - Responsibility for health and social care for older people outside of hospital was transferred to local municipal government |
| **41** | **Denmark cross-charging**  [90] | Denmark |  | Cross charging | Health  Social Care | Individuals in acute care |  | **Evaluation methodology:** No evaluation identified  **Outcome affect:**  **Process affect:** |
| **42** | **Somerset Partnership Health and Social Care Trust**  [91]; [92]; [93]; [94]; [95]; [96]; [97] | England | Combined provision of mental health & social care services. | Joint commissioning | Health  Social Care | Individuals with mental health issues | NHS Plan (2000); Health Act (1999); NHS Act (2006) | **Evaluation methodology**: Quantitative (trend); Qualitative (interviews, focus group discussion, surveys)  **Outcome affect:**   - Some reported improvement in self-reported mental health status. - Largely no observed benefits.   **Process affect:** |
| 43 | **Community Health Partnerships (CHPs) / Community health and care partnerships (CHCPs)**  [98,99] | Scotland | Primary health care and social services | Aligned budgets | Health  Social Care | Whole local population covered |  | **Evaluation methodology:**  Qualitative (interviews, survey)  O**utcome affect:**   - Poor effect on reducing health inequalities and reducing death in preventable diseases. - Slight perceived beneficial effect on waiting times and avoidable hospital admission reductions   **Process affect:** |
| 44 | **Pilot of Partnerships for Older People Projects**  [100] | England | Community interventions aimed at elderly.  Specific interventions varied by project with 29 Local Authority led sites running 146 projects. | Pooled budgets | Health  Social Care | Elderly | - | **Evaluation methodology:**  Qualitative (interviews, focus group discussion, document review etc.)  Quantitative (quasi-experimental methods)  **Outcome affect:**   - Participants self-reported improved HRQoL and access to services.   **Process affect:**   - Difference-in-difference shows significant reduction in emergency bed day use: - Large cost savings seen from programme compared to controls |
| 45 | **North East Lincolnshire Care Trust Plus**  [101]; | England | Learning disability, community equipment and mental health services | Pooled budget | Health  Social Care | Whole local population covered | NHS Plan (2000); Health Act (1999); NHS Act (2006) | **Evaluation methodology :**See Care Trusts  **Outcome affect:**  **Process affect:** |
| 46 | **Torbay Care Trust**  [102]; | England | Health & social care services | Full organisational & budgetary integration | Health  Social Care | Whole local population covered | NHS Plan (2000); Health Act (1999); NHS Act (2006) | **Evaluation methodology:** Qualitative  Outcome affect:  Process affect: |
| 47 | **Wye Valley NHS Trust**  [30]; [18]; [103] | England | Integrated acute, community and adult social care including services such as integrated community equipment services (ICES) store | Full organisational & budgetary integration | Health  Social Care | Whole local population covered | NHS Plan (2000); Health Act (1999); NHS Act (2006) | **Evaluation methodology:**Qualitative  **Outcome affect:**   - Self-reported efficiency savings - Reduction in delayed discharges and 1,100 bed days saved.   **Process affect:** |
| 48 | **Evaluation of Integrated Resource Framework Test Sites**  [14]; [104] | Scotland | Long term care for older individuals, individuals with chronic conditions and disabilities or mental illness. | Full organisational & budgetary integration | Health  Social Care |  |  | **Evaluation methodology:** Qualitative (document review, interviews, focus group discussion).  **Outcome affect:**  **Process affect:** |
| 49 | **The Norrtalje Model**  [105] | Sweden |  | Full organisational & budgetary integration | Health  Social Care |  |  | **Evaluation methodology:** Qualitative (document review, interviews)  **Outcome affect:**   - Simulation model comparing observed and expected nursing home stays and costs suggest may be cost-saving.   **Process affect:** |
| 50 | **Arizona Long Term Care System**  [106] | United States | Health & social care services | Pooled budgets | Health  Social Care | Individuals with severe physical and developmental impairment |  | **Evaluation methodology:** Quantitative (trend)  **Outcome affect:**  **Process affect:** |
| 51 | **Commonwealth Care Alliance**  [107] | United States | Co-ordination of primary, mental health and social care in the community by multidisciplinary teams | Pooled budgets | Health  Social Care | Elderly, young people with physical and mental disabilities, adults and children with multiple chronic illnesses |  | **Evaluation methodology:** Quantitative (trend)  **Outcome affect:**   - Lower bed days, rate of nursing home placement - Reduced total health care spending growth.   **Process affect:** |
| 52 | **Community Medical Alliance (CMA)**  [108]; [109] | United States | Mental health services, long-term care and social services | Pooled budgets | Health  Social Care | Disabled individuals |  | **Evaluation methodology:** Quantitative (trend)  **Outcome affect:**  Reduced per capita and acute hospital spend for individuals with physical disability  **Process affect:** |
| 53 | **Minnesota Senior Health Options (MSHO)**  [110]; [111]; [112]; [113]; [114]; [115] | United States | Health and long-term care services | Pooled budgets | Health  Social Care | Individuals aged 65+ eligible for Medicare & Medicaid |  | **Evaluation methodology**: Quantitative (quasi-experimental)  **Outcome affect:**  **Process affect:** |
| 54 | **On Lok**  [116]; [117] | United States | Provision of health and social care services in adult day care | Pooled budgets | Health  Social Care | Elderly |  | **Evaluation methodology:** Quantitative (quasi-experimental)  **Outcome affect:**   - Improvements in functional independence - Participants costs per person were 21% lower than control group due to lower costs of inpatient care   Process affect: |
| 55 | **Social Health Maintenance Organisations**  [118]; [119]; [110]; [120]; [121]; [122]; [123] | United States | Health and social care services | Full organisational & budgetary integration | Health  Social Care | Elderly |  | **Evaluation methodology**: Quantitative (quasi-experimental)  **Outcome affect:**   - Reduced satisfaction and higher mortality rate compared to control group - Higher nursing home and home care costs with corresponding lower hospital costs   **Process affect**: |
| 56 | **Veterans Health Administration**  [41][124]; [125]; [126])[127];[128] | United States | Health & social care services | Pooled budgets | Health  Social Care | Disabled veterans |  | **Evaluation methodology:** Quantitative (quasi-experimental)  **Outcome affect:**   - Fall in hospital admissions, bed days expenditure   **Process affect:** |
| 57 | **Wisconsin Partnership Program (WPP)**  [111]; | United States | Health and social care services | Pooled budgets | Health  Social Care | Elderly |  | **Evaluation methodology**: Quantitative (trend)  **Outcome affect:**   - No significant differences in mortality, hospital admission rates, length of stay, preventable hospital admission rates, and use of emergency services.   **Process affect:** |
| 58 | **Access to Community Care and Effective Services and Supports (1994-1998)**  [129–134] | United States | Provided funds and technical assistance to nine community sites to im- plement strategies for system change that would promote systems inte- gration.  System change strategies for integrating mental health, substance abuse, housing, primary care, and income maintenance services. | Grant for applications | Health Mental health Housing Rehabilitation | Individuals with multiple social and health care needs |  | **Evaluation methodology:** RCT  **Outcome affect:**   - Better systems integration did not lead to improvements in individual outcomes   **Process affect:**  Experimental sites did have higher levels of system integration across sectors involved Ferguson, et al., 2012  interviews with key informants from relevant or- ganizations in each community. Client outcome data were obtained at program entry and three and 12 months later from 7,055 program par- ticipants across the four annual client cohorts at all sites. |

**Table 2: Promotion Cases**

| **No** | **Co-financing case (year)** | **Country** | **Co-financed intervention** | **Financial mechanism** | **Payers involved** | **Target population** | **Legal framework** | **Evaluation** |
| --- | --- | --- | --- | --- | --- | --- | --- | --- |
| **1** | **School Health & Nutrition**  [135,136]; [137] | Zambia | School-based deworming, micro nutrition & health education | In-kind support  (education infrastructure & human resources) | Health – JICA; Gates Foundation  Education – USAID Education Programme; Ministry of Education | Children | Joint MOU & implementation guidelines | Evaluation Methodology: Quantitative (RCT)  Outcome effect:  ***Health***   - Prevalence of parasitic worm infection reduced by 75% from baseline - *Education* - Significant & cumulative increase in cognitive test scores   **Process effect:**   - National School Health & Nutrition Policy (2006) developed |
| **2** | **Road Safety Partnership Grant**  [138–140] | England | Intersectoral projects improving road safety | Grant requiring intersectoral proposals & additional partner financing | Health  Transport  Education  Justice | National population | The Road Safety Act (2006) | Evaluation Methodology: Qualitative (self-reporting narratives, document review), overall assessment & individual assessment of projects  **Outcome effect:**  *Health*   - Sample of projects costing £6m resulted in estimated annual casualty reduction benefits of £11.5m – a 190% return on investment   **Process effect:**   - Total of 56 projects approved (2007-11) |
| **3** | **South Australia Health in All Policies - Health Lens Analysis Projects**  [141–145] | Australia |  |  | Health |  |  | **Evaluation methodology:**  **Outcome effect:**  **Process effect**: |
| **4** | **Geracão Biz Program (PGB)**  [146]; [147]; [53,148] | Mozambique | Multi-sectoral adolescent sexual & reproductive health programme | Aligned funding | - Health – Ministry of Health - Education – Ministry of Education - Youth – Ministry of Youth and Sports | Youth & adolescents |  | **Evaluation Methodology**: Quantitative (trend); Qualitative (interviews, document review)  **Outcome effect:**   - *Health* - 1,503% increase in youth service utilisation in Maputo (1999-2001) - 57% of PGB exposed youth used contraceptive compared to 53% of non-PGB exposed youth (2011)   **Process effect:**   - Positive perception of services among youth |
| **5** | **New York City Childhood Asthma Initiative**  [149]; [150]; | United States | Community education on asthma and case coordination | Grant requiring intersectoral proposals & additional partner financing | - Health – Department of Health and Mental Hygiene | Children |  | **Evaluation Methodology:** Quantitative (trend)  **Outcome effect:**   - *Health* - Reduction in hospitalization rates from 9.43 children to 6.06, 35% reduction, between 1997-2000 in NYCCAI neighbourhoods - Evaluations to date of the home-based education component of the program have indicated a positive impact on several health outcomes   **Process effect:**   - Numerous asthma projects created such as East Harlem Asthma Centre for Excellence & Managing Asthma in Schools |
| **6** | **Kenya National School-Based Deworming Programme**  [151] ; [151–153] [154] | Kenya | School-based deworming | In-kind support (education infrastructure & human resources) | Health – Ministry of Health (GlaxoSmithKline  and Merck donated drugs)  Education – Ministry of Education | Children | National School-Based Deworming Programme (2009) | **Evaluation Methodology:** Quantitative (trend); Qualitative (interviews, focus groups)  **Outcome effect:**   - *Health* - 73% & 29% reduction in prevalence of STH & Schistosomiasis respectively in 1^st^ year - 51% reduction in STH pre-Y3 MDA compared to pre-Y1 MDA   **Process effect:**   - 5.99 million school-aged children treated in 2012/3 - 6.4 million school-aged children treated in 2013/4 - 6.17 million school-aged children treated in 2014/5 |
| **7** | **District of Columbia Mayor’s Council on Physical Fitness, Health, and Nutrition** **Fitness Fund**  [155–157] | United States | Convenes health, education & recreation & parks depts. to advise on obesity-related objectives with separate ring-fenced Fitness Fund | Pooled budget  (at source) | District Treasury – Fitness Fund | Regional population | Mayor’s Council on Physical Fitness, Health and Nutrition Act (2011) | **Evaluation Methodology:** No evaluation identified  **Outcome effect:**  **Process effect:** |
| **8** | **Prince Edward Island**  [158] [159]; [160] | Canada | Pooled budget encouraging cross-sectoral resource reallocation to address determinants of health | Pooled budget (block grants to regions for bundle of human services) | Health  Education  Recreation & parks  Housing  Probation & correctional services  Child welfare  Income security  Employment | Regional population | Health and Community Services Act (1993) | **Evaluation Methodology**: Qualitative (interviews, focus groups, document review)  **Outcome effect:**  **Process effect:**   - Overall cautious approach to actual reallocation despite enthusiasm for concept - 74 identified examples of resource reallocation (mostly within health care sub-sectors or social care sub-sectors) - Few examples of reallocation from acute care to social determinants or community-based care - Reallocations among ‘real’ resources (staff, equipment, supplies etc.) more common than financial resource reallocation |
| **9** | **Justice Sector Fund**  [161–163] | New Zealand | Seed funding for projects aimed at impacting interagency performance targets | Pooled budget (for projects approved by the justice sector board) | Police – New Zealand Police  Justice – Ministry of Justice  Corrections – Department of Corrections  Fraud – Fraud Office | National population | Amendment to Public Finance Act (2013) including Cross-Agency Funding Framework (2014) | **Evaluation Methodology**: Individual project evaluations  **Outcome effect:**  *Police*   - 34-percent reduction in youth crime at the end of 2016.   **Process effect:**   - 66 projects funded between 2012-17 costing NZ$273M |
| **10** | **Virginia Children’s Services Act**  [164];[165]; [166–169] | United States | Cross sector activities for at-risk youth & families including education, housing, physical & mental health, transport & food | Pooled budget  (blended) | Social services – Department of Social Services  Education – Department of Education  Justice – Department of Juvenile Justice  Health – Department of Mental Health, Mental Retardation, and Substance Abuse | At-risk youth & families | Virginia General Assembly Comprehensive Services Act (1992) renamed Children’s Services Act (2015) | **Evaluation Methodology**: Not found  **Outcome effect:** Not found  **Process effect:** Not found |
| **11** | **Massachusetts Health Policy Commission Health Care Innovation Investment Program - The 10th** **Decile Project**  [165,170]; | United States | Provision of homeless with health, housing & social services | Grant requiring intersectoral proposals | Health – 18 partner hospitals; Homeless Health Care Los Angeles; San Fernando  Community Mental Health  Housing – LA Family Housing; Housing Works;  Justice | Homeless individuals |  | **Evaluation Methodology**: Quantitative (trend, matching)  **Outcome effect:**  *Housing*   - 44 individuals received housing vouchers, 36 were received permanent supportive housing, 7 individuals died and 5 individuals were incarcerated after program contact.   *Health*   - For those enrolled and moved to housing total health care costs decreased by 72% per person (from $58,962 to $16,474)   *All*  $1 spent on the program resulted in $2 net saving in 1^st^ year and $6 for every subsequent year  **Process effect:**   - 163 homeless individuals screened with 89 enrolled in the program. - 12 referral sources (hospitals, medical centres |
| **12** | **Programme for the Modernisation of Agriculture (PMA)**  [20,171–173] | Uganda | Activities in any sectors which fall under 7 pillars of the PMA | Aligned budget; non-sectoral conditional grants | Agriculture – Ministry of Agriculture, Animal Industry and Fisheries; National Agricultural Research Organisation  Water & sanitation – Ministry of Water, Lands and Environment  Tourism – Ministry of Tourism, Trade and Industry  Education – Ministry of  Education and Sports  Health – Ministry of Health  Poverty & Economic Development – Ministry of Works, Housing and Communications; Ministry of Local Government; Ministry of Finance, Planning and Economic Development  Gender – Ministry of Gender, Labour and Social Development |  |  | **Evaluation Methodology:** Qualitative (stakeholder consultation, document review, qualitative household survey, district visits, focus groups)  **Outcome effect:**  *All*   - Estimated economic return of National Agricultural Advisory Services of 18%   *Agriculture*   - Most farmers surveyed reported that yields had either decreased or remained constant but felt livestock production had increased   *Economic Development*   - Self-reported relative community wealth ranking showed members of farmers groups felt better off   **Process effect:**   - PMA accounts for 10% of Government spending - 155 project identified at PMA relevant between 2001-04 - Only 54% of budgeted expenditure for PMA was disbursed between 2001-04 (disbursement rate worse among donors than Gov. depts.) - PMA pillars implemented at different rates with varying success and not as pro-poor as hoped - Some ministries supposed to be active in the PMA are unaware or uncertain of its purpose - Membership in PMA associated farmers groups varied by district sampled between 10-42% |
| **13** | **York Pathways Pilot**  [174] | England | Mental health support for individuals at-risk of entering criminal justice system | Pooled budget | - Police – North Yorkshire Police - Health – Clinical Commissioning Group - Social services – County Council | Young adults in contact with police |  | **Evaluation Methodology**: Qualitative (stakeholder interviews)  **Outcome effect:**  *Police*   - Reported decrease in police call-outs   **Process effect:** |
| **14** | **Transition to Adulthood (T2A) Together for Mental Wellbeing** programme  [175,176] | England | Mental health support for individuals at-risk of entering criminal justice system |  | - Police – South Yorkshire Police - Health – Clinical Commissioning Group | Young adults in contact with police |  | **Evaluation Methodology:** Quantitative (trend); Qualitative (document review, stakeholder interview)  **Outcome effect:**   - *Police* - Reduced young person contact with police by 44% (comparison of pre and during intervention trends) - Positive user testimonials   **Process effect:**   - 52 cases between 2014-2017 out of an intended 120 cases - 230 day average duration of engagement |
| **15** | **Ceará Multi-Sector Social Inclusion Development Program**  [177–181]; [138–140] | Brazil | Funding upstream interventions to ensure all sector targets met | Combination of World Bank SWAp & ALP funds disbursed to State Treasury supporting 9 budget programmes across 6 ministries with disbursement based on cross-sector indicators (implicit cross-charging component) | Education  Environment  Health  Water & Sanitation  Water Resource Management  Public Sector Management | Regional population | Ceara Loan Agreement (2005) | **Evaluation Methodology**: Quantitative (trend); Qualitative (stakeholder consultations)  **Outcome effect:**  *Education*   - Illiterate population aged 15+ reduced by 100,000   *Health*   - 90%+ women receiving 4+ pre-natal visits - 60%+ population covered by Family Health Program teams - *Water and Sanitation* - 34,160 new household water connections   **Process effect:**   - The SWapL forged collaboration between line and central ministries - US$149.8M disbursed by 2008 (100% intended disbursement) - World Bank assessment report outlined Bank and Borrower performance as satisfactory (2008) - Loan model replicated in several other Brazilian states - Of the 23 indicators required for the 4^th^ disbursement, 13 were exceeded, 7 were complied with & 3 experienced difficulties - Loan met the majority of the conditionality triggers for the 2^nd^ phase - Implanted culture of results-based management - Leveraged financing across the 6 ministerial sectors |
| **16** | **The Berks County Community Prevention coalition**  [182] | United States | Broad range of substance abuse prevention programmes |  | Addiction – Pennsylvania Liquor Control Board; SAMHSA’s Center for Substance Abuse Prevention; Council On Chemical Abuse  Justice – U.S. Department of Justice’s Office of Juvenile Justice and Delinquency Prevention; Governor's Partnership for Safe Children/Pennsylvania Commission on Crime & Delinquency  Housing – Reading Housing Authority  Social services – Pennsylvania Department of Public Welfare; Berks County Intermediate Unit |  |  | **Evaluation Methodology:**  **Outcome effect:**  **Process effect**: |
| **17** | **The Interagency Program for the Empowerment of Adolescent Girls (IPEAG)**  ((MSPAS)) [183] | El Salvador | Programme providing an integrated response to the needs of adolescent girls | Aligned budgets | Health – Pan American Health Organisation (through Ministry of Health); UNDP  Education – UNICEF (through Ministry of Education)  Agriculture – Food and Agriculture Organisation; UNFPA | Adolescent girls |  | **Evaluation Methodology:** Qualitative (stakeholder consultations, document review, semi-structured interviews)  **Outcome effect:**  **Process effect**:   - Reached 28,573 adolescent girls with integrated health care including SRH between 2005-06 |
| **18** | **Better Beginnings, Better Futures (BBBF) project**  **(1990)**  [161]; [184] [[179} | Canada | Community-based mental health promotion | Grants with request for proposals | Social services – Ministry of Community and Social Services  Health – Ministry of Health  Education – Ministry of Education and Training | Children & families |  | **Evaluation Methodology:** Quantitative (matching); Qualitative (interviews)  **Outcome effect:**  *All*   - Programme had net benefit of US$3,777 (2010) per family - Economic return of $2.50 for every $1 invested - Improved social functioning of participants   *Education*   - Average grade increase of 2% by grade 12 of participants - Lower proportion using special education services in high school (15% compared to 23%)   *Health*   - Lower proportion of parents with clinical depression (18% compared to 27%)   **Process effect:**   - Schools became hubs for seamless delivery of array of services - Greater level of service integration through enhanced partnerships - 20 years of research identifying Better Beginnings as a successful & cost-effective initiative led to dissemination of findings across Canada |
| 19 | **Office of Northamptonshire Police and Crime Commissioner early intervention to support mental health** [185] | England |  |  | Justice – Northamptonshire Police and Crime Commissioner |  |  | **Evaluation Methodology:** No evaluation identified  **Outcome effect:**  **Process effect:** |
| **20** | **National Development Programme for Social Welfare and Health Care (Kaste programme)**  **(2008-2015)**  [186–190]; [191] | Finland | Delivery of intersectoral health promotion activities promoting  physical, mental and social wellbeing and preventing problems  across the entire population. | Grant for intersectoral activities | Health – The National Institute for Health and Welfare (THL)  (health is the only payer)  Social Care – Finnish Institute of Occupational Health (TTL)  Education |  | Act on Planning and Government Grants for Social Welfare and Health Care | **Evaluation Methodology:** Qualitative (interviews)  **Outcome effect:**  **Process effect:**   - €17.5 million per annum is allocated to the programme - Actual disbursements from the Ministry of Social Affairs & Health between 2012-2015, for Kaste projects totalled about €46 million - 34 development projects complying with the programme and aimed at reforming and developing the social welfare and health care services were granted transfers |
| **21** | **Varde Fund for Health**  **(2007)**  [192,193]; | Denmark | Intersectoral activities that aimed at improving health and the quality of life, and to make the ‘healthy choice the easy choice’ for all citizens. | Grant for intersectoral activities (Local Authority held funds and any council department could apply for access. Award for funding conditional on involvement of two or more sectors) | Health – Local Authority | Local population |  | **Evaluation Methodology:** Qualitative (interviews, document review)  **Outcome effect:**  **Process effect:**   - initial earmarked budget of 1 million Danish kroner (US$200,000) - As well as creating health networks to share information and knowledge, the fund helped to stimulate intersectoral activity and overcome budgetary silos. It is considered a model for wider adoption in other municipalities. |
| **22** | **Population Health Fund (PHF) of the Public Health Agency of Canada**  **(1997-2008)**  [194]; [195] | Canada | Advance approaches to address the social determinants of health using intersectoral action e.g. a four-year project to improve nutrition in children and their parents through collaboration among child care centres and schools, food retailers, the agricultural sector | Grant for intersectoral activities (Award for funding conditional on involvement of two or more sectors) | Health – Public Health Agency of Canada | Local population |  | **Evaluation Methodology:** Qualitative (document review, interviews, survey), overall assessment & individual assessment of projects  **Outcome effect:**   - *Economic Development*   The ‘Garderie Bio: Manager et Grandir’ project linked organic farms to child centres improving children’s diet and farmers income  *Health*   - 7% of projects reported that they had contributed to positive changes in health outcomes   **Process effect:**   - Approximately Can$ 12.1 million per annum was divided equally between national and regional projects. - Each regional project ran for an average of 26 months and received Can$ 150,000, while national projects ran for 36 months on average and received Can$ 312,000. - 116 projects completed between 2005-2008 - more than a quarter of successful applications were able to provide/raise additional funding, while 54% provided additional in- kind resources |
| **23** | **Innovation Strategy Fund (Replaced PHF)**  **(2009 )**  [196] | Canada | deliver innovative population health interventions to reduce health inequalities and address priority complex public health problems and their underlying factors (particularly activities tackling mental health issues and obesity) | Grant for intersectoral activities (often includes matching funds from applicant organisations) | Health – Public Health Agency of Canada | Local population |  | **Evaluation Methodology:** Qualitative (interviews, survey, case studies)  **Outcome effect:**  *Health*   - 78% of projects reported that they had contributed to positive changes in health outcomes - In one project funded, participants 80% of participants report making healthy changes to their diet, 69% report eating more fruits and vegetables, 80% report improvement in their mental health, and 55% said their physical health has improved   **Process effect:**   - New funding model had more success in developing sustainable population health interventions - Program budget of CAN$54 million over five years - Approximately CAN$ 9.7 million per year awarded to successful project applicants between 2009-2014 - Partnerships developed through Innovation Strategy enabled projects to raise approx. CAN$ 5.7 million in additional funds and obtain approximately CAN$ 5.6 million of in-kind support - Leveraged funds have most often been received from other federal government departments, P/T governments, regional health authorities, not-for-profit organizations and universities - 52 projects received phase 1 funding with 20 continuing on to receive phase 2 funding - Projects in phase 1 reached 89,000 individuals between 2009-2011. - Projects in phase 2 reached 550,000 individuals |
| **24** | **Wonju Healthy City Project**  **(2004)**  [197,198]; | Republic of Korea | Intersectoral health promotion activities | Earmarked tax (sin tax on tobacco consumption) | Health | Local population | bill allocating all revenues from the tobacco consumption tax to the Healthy City Wonju project for the following 5 years. | **Evaluation Methodology:** Qualitative (document review)  **Outcome effect:**  *Health*   - Awarded six times by WHO - Smoking rate fell from 57.8% for males and 6.8% for females in 2004 to 46.9% for males and 3.4% for females in 2008   **Process effect:**   - The project initially had an annual budget in 2006 of US$16 million - In 2006, 66 programs implemented under the project. Programmes including creating smoke-free zones, life-stage and setting-specific (e.g. schools, workplaces, hospitals) health promotion strategies, greening the city and improving housing conditions - Budget allocation were ‘education for children and the youth’ (a quarter), ‘healthy lifestyles (exercise, sobriety, non-smoking)’ (18%), ‘culture and welfare’ (17%) and ‘food and nutrition’ (16%) |

**References**

1. Stokes J, Lau Y-S, Kristensen SR, Sutton M. Does pooling health & social care budgets improve quality and lower costs? 2017;20.

2. Better Care Fund: policy framework. :13.

3. Department of Health, Department for Communities, Local Government. Health and social care integration [Internet]. 2017 p. 56. Available from: https://www.nao.org.uk/wp-content/uploads/2017/02/Health-and-social-care-integration.pdf

4. Audit Commission for Local Authorities. Means to an end: joint financing across health and social care: health national report [Internet]. England: NHS; 2009. Available from: https://www.bl.uk/britishlibrary/~/media/bl/global/social-welfare/pdfs/non-secure/m/e/a/means-to-an-end-joint-financing-across-health-and-social-care-health-national-report.pdf

5. Humphries R, Wenzel L. Options for integrated commissioning. 2015;64.

6. Social Care, Ageing and Disability. Integration and Better Care Fund Policy Framework 2017 to 2019 [Internet]. London, UK: Department of Health; 2017. Available from: https://assets.publishing.service.gov.uk/government/uploads/system/uploads/attachment_data/file/607754/Integration_and_BCF_policy_framework_2017-19.pdf

7. Humphries R, Wenzel L. Options for integrated commissioning. :64.

8. Turner AJ, Urwina S, Sutton M. INITIAL IMPACTS OF HEALTH AND SOCIAL CARE DEVOLUTION IN GREATER MANCHESTER: EFFECTS ON PRIMARY CARE PRESCRIBING. 2017.

9. Ainsworth M. Working Well Expansion. Manchester: GM Lead for Employability Initiatives. 2016.

10. Pike B, Mongan D. The integration of health and social care services. :137.

11. Hendry A. Creating an Enabling Political Environment for Health and Social Care Integration. International Journal of Integrated Care [Internet]. 2016 [cited 2019 Mar 15];16. Available from: http://www.ijic.org/articles/10.5334/ijic.2531/

12. Government TS. National Health and Wellbeing Outcomes: A framework for improving the planning and delivery of integrated health and social care services. The Scottish Government. 2015;

13. Ham C, Heenan DA, Longley M, Steel DR. Integrated care in Northern Ireland, Scotland, and Wales: lessons for England. 2013.

14. NHS Scotland. Reshaping Care for Older People - A Programme for Change 2011-2021. UK: NHS Scotland; 2011 p. 16.

15. Harris J, Nguyen P, To Q, Hajeebhoy N, Phan L, Vu H, et al. Improvement in provincial plans for nutrition through targeted technical assistance and local advocacy in Vietnam. FASEB Journal Conference: Experimental Biology. 2015;29.

16. Team JI. Reshaping Care for Older People Change Fund: Building on Progress. Scotland: NHS; 2015.

17. Reshaping care for older people - Impact report. :16.

18. Audit Commission for Local Authorities. Means to an end: joint financing across health and social care: health national report [Internet]. England: NHS; 2009. Available from: https://www.bl.uk/britishlibrary/~/media/bl/global/social-welfare/pdfs/non-secure/m/e/a/means-to-an-end-joint-financing-across-health-and-social-care-health-national-report.pdf

19. Miller R, Dickinson H, Glasby J. The vanguard of integration or a lost tribe? Care trusts ten years on. Health. 2011;

20. Welle K, Tucker J, Nicol A, Evans B. Is the water sector lagging behind education and health on aid effectiveness? Lessons from Bangladesh, Ethiopia and Uganda. Water Alternatives. 2009;2:297–314.

21. Evans D, Forbes T. Partnerships in Health and Social Care: England and Scotland Compared. Public Policy and Administration. 2009;24:67–83.

22. Curry N, Harris M, Gunn L, Pappas Y, Blunt I, Soljak M, et al. Integrated care pilot in north west London: a mixed methods evaluation. International Journal of Integrated Care [Internet]. 2013 [cited 2019 Mar 15];13. Available from: http://www.ijic.org/article/10.5334/ijic.1149/

23. Wistow G, Waddington E. Learning from Doing: Implications of the Barking and Dagenham Experience for Integrating Health and Social Care. Journal of Integrated Care. 2006;14:8–18.

24. Health NP. National Evaluation Of Notifications for Use of the Section 31. Partnership Flexibilities in the Health Act 1999 Final Project Report. 2002.

25. Dickinson H. Evaluating the outcomes of health and social care partnerships: the POET approach. Research, Policy and Planning. 2007;25:79–92.

26. Hudson B. Policy into Practice Partnership Working between Health and Social Care: the Health Act 1999. 2004;22:6.

27. Sillett J. Clarifying joint financing arrangements - A briefing paper for health bodies and local authorities [Internet]. Audit Commission; 2008. Available from: http://www.yor-ok.org.uk/CommissioningNatDocs/JointFinancing4Dec08.pdf

28. Roland M, Lewis R, Steventon A, Abel G, Adams J, Bardsley M, et al. Case management for at-risk elderly patients in the English integrated care pilots: observational study of staff and patient experience and secondary care utilisation. International Journal of Integrated Care [Internet]. 2012 [cited 2019 Mar 15];12. Available from: http://www.ijic.org/article/10.5334/ijic.850/

29. RAND Europe, Ernst and Young. National Evaluation of the Department of Health’s Integrated Health Pilots [Internet]. UK: RAND Europe; 2012. Available from: https://www.rand.org/content/dam/rand/pubs/technical_reports/2012/RAND_TR1164.pdf

30. Licence N, Gurney F. Health and Social Care integration has drastically reduced delayed discharge in Herefordshire [Internet]. Herefordshire: NHS Trust Wye Valley; 2011. Available from: https://www.wyevalley.nhs.uk/media/11689/51-11DelayedDischarge250711.pdf

31. Challis D, Darton R, Johnson L, Stone M, Traske K. An Evaluation of an Alternative to Long-stay Hospital Care for Frail Elderly Patients: II. Costs and Effectiveness. Age and Ageing. 1991;20:245–54.

32. Freeman T, Peck E. Evaluating partnerships: a case study of integrated specialist mental health services. Health and Social Care in the Community. 2006;14:408–17.

33. Hudson B. Joint commissioning across the primary health care-social care boundary: Can it work? Health and Social Care in the Community. 1999;7:358–66.

34. Soljak M, Cecil E, Gunn L, Broddle A, Hamilton S, Tahir A. Quality of care and health outcomes. London : Imperial College London. 2013;

35. Steeden A. The Integrated Care Pilot in North West London. London Journal of Primary Care. 2013;5:8–11.

36. Bardsley M, Smith J, Car J. Evaluation of the first year of the Inner North West London Integrated Care Pilot [Internet]. Nuffield: Imperial College of London; 2013. Available from: https://www.nuffieldtrust.org.uk/files/2017-01/evaluation-inner-north-west-london-integrated-care-pilot-web-final.pdf

37. Greaves F, Pappas Y, Bardsley M, Harris M, Curry N, Holder H, et al. Evaluation of complex integrated care programmes: the approach in North West London. International Journal of Integrated Care [Internet]. 2013 [cited 2019 Mar 15];13. Available from: http://www.ijic.org/article/10.5334/ijic.974/

38. <Transforming the NHS in North West London Integrating health and social care with the leadership of local GPs and working in partnership with NHS England.pdf>.

39. Roberts D. Operating a Pooled Budget and Lead Commissioning Using Health Act Flexibilities: The Oxfordshire Experience. Journal of Integrated Care. 2006;14:36–44.

40. Chapin RK, Wendel C, Lee R, Landry S, Zimmerman MK, Oslund P, et al. Program of All-inclusive Care for the Elderly (PACE) Medicaid Cost-Benefit Study [Internet]. Kansas: Kansas Department for Ageing and Community Services; 2013. Available from: https://www.npaonline.org/sites/default/files/PDFs/PACE%20Final%20Report_KS.pdf

41. Curry N, Ham C. Clinical and service integration The route to improved outcomes. London: The King’s Fund; 2010.

42. Nyström ME, Strehlenert H, Hansson J, Hasson H. Strategies to facilitate implementation and sustainability of large system transformations: a case study of a national program for improving quality of care for elderly people. BMC Health Services Research [Internet]. 2014 [cited 2019 Mar 15];14. Available from: https://bmchealthservres.biomedcentral.com/articles/10.1186/1472-6963-14-401

43. NHS Department of Health. Guide to Integrating Community Equipment Services. UK: NHS Department of Health; 2001.

44. Awes A, Rom M, Askenbom P, Kleinman C, Sveriges kommuner och landsting. A better life for elderly sick people: major improvments over a short time. Stockholm: Sveriges kommuner och landsting; 2015.

45. O’Brien M, Bachmann MO, Jones NR, Reading R, Thoburn J, Husbands C, et al. Do integrated children’s services improve children’s outcomes?: Evidence from england’s children’s trust pathfinders. Children and Society. 2009;23:320–35.

46. Hultberg E-L, Glendinning C, Allebeck P, Lonnroth K. Using pooled budgets to integrate health and welfare services: a comparison of experiments in England and Sweden. Health and Social Care in the Community. 2005;13:531–41.

47. Löfström M. Inter-organizational collaboration projects in the public sector: A balance between integration and demarcation. International Journal of Health Planning and Management. 2010;25:136–55.

48. Insurance C o. Finsam – a follow-up of nancial coordination of rehabilitation measures. Sveriges Riksdag; 2014.

49. Hultberg E-L, Glendinning C, Allebeck P, Lonnroth K. Using pooled budgets to integrate health and welfare services: a comparison of experiments in England and Sweden. Health and Social Care in the Community. 2005;13:531–41.

50. Allebeck P. Mapping Household-Based Health Security - The Case of Sweden. Social Theory and Health. 2008;6:61–73.

51. Wihlman U, Lundborg CS, Axelsson R, Holmström I. Barriers of inter-organisational integration in vocational rehabilitation. International Journal of Integrated Care [Internet]. 2008 [cited 2019 Mar 15];8. Available from: http://www.ijic.org/article/10.5334/ijic.234/

52. Alexanderson K, Norlund A. Swedish Council on Technology Assessment in Health Care (SBU). Chapter 1. Aim, background, key concepts, regulations, and current statistics. Scand J Public Health Suppl. 2004;63:12–30.

53. Berry C, Kaplan SA, Reid A, Albert S. The Viability of Community Partnerships Initiated by External Funders. Public Health Reports. 2009;124:590–3.

54. AMCHP. Oregon Youth Transition Program [Internet]. Oregon: Association of Maternal and Child Health Programs; 2011. Available from: http://www.amchp.org/programsandtopics/BestPractices/InnovationStation/ISDocs/Oregon%20YTP_2015.pdf

55. ABT Associates. Home For Good Funders Collaborative: Lessons Learned from Implementation and Year One Funding. California, USA: ABT Associates; 2013 May.

56. Brown J, Wilkins C, Fiore N, de Sousa T, Savidge G, Spellman B. Evaluation of the Conrad N. Hilton Foundation Chronic Homelessness Initiative - Phase I Final Report [Internet]. ABT Associates; 2016. Available from: https://www.hiltonfoundation.org/learning/chronic-homelessness-initiative-2016-evaluation-report-phase-i

57. Bomba J. Home for Good: An initiative of United Way of Greater Los Angeles & L.A. Area Chamber of Commerce: The Action Plan to End Chronic and Veteran Homelessness by. Home For Good United Way of Greater Los Angeles; 2014.

58. Associates A. Home For Good Funders Collaborative: Updated Lessons Learned from Five Years of Coordinated Funding. Abt Associates Inc; 2017.

59. Briefing Paper: The Community Sexual Violence Sector in the Auckland Region. Auckland, NZ: New Zealand Family Violence Clearinghouse; 2011.

60. Alper J, Thompson D, Baciu A. Exploring Opportunities for Collaboration Between. National Academy of Sciences. 2015;

61. Flynn M, Hayes C. Blending and Braiding Funds To Support Early Care and Education Initiatives [Internet]. New York: The Finance Project; 2003 Jan. Available from: file:///C:/Users/Admin/Downloads/Lavanya%20References/1a%20MISSING%20LITERATURE/Blending%20and%20Braiding%20Funds%20To%20Support%20Early%20Care%20and%20Education%20Initiatives%20Flynn%20&%20Hayes%20(2003).pdf

62. Learning MD. Policy Brief: Increasing Early Childhood Programs Through Blended and Braided Funding. 2011.

63. Early Care and Child Consortium. New Mexico Policy Facts - Blending and Braiding Funding to Support High Quality Child Care [Internet]. New Mexico, US: Early Care and Education Consortium; 2013 Jan. Available from: http://www.ececonsortium.org/wp-content/uploads/2014/01/New-Mexico-Early-Brain-Science-Early-Childhood-Policy-Dev-Jan-13.pdf

64. DISABILITY NC. Blending and Braiding Funds and Resources: The Intermediary as Facilitator. 2006.

65. Joint Commissioning Strategy for Speech & Language Therapy for children & young people in Enfield [Internet]. 2011. Available from: https://governance.enfield.gov.uk/Published/C00000116/M00006609/AI00019055/$SALTJointCommissioningStrategy20102013V4.docA.ps.pdf

66. Guildford and Waverly Clinical Commissioning Group. Joint Commissioning Strategy for Speech and Language Therapy Services for Children and Young People in Surrey 2014 - 2017 [Internet]. Surrey County Council; 2014. Available from: https://www.surreycc.gov.uk/__data/assets/pdf_file/0006/85920/SLT-Joint-Commissioning-Strategy_v0.2.pdf

67. National Audit Office UK. Healthcare across the UK- A comparison of the NHS in England, Scotland, Wales and Northern Ireland [Internet]. 2012. Available from: https://www.nao.org.uk/wp-content/uploads/2012/06/1213192.pdf

68. Heenan D, Birrell D. Organisational Integration in Health and Social Care: Some Reflections on the Northern Ireland Experience. Journal of Integrated Care. 2009;17:3–12.

69. Battersby MW. Health reform through coordinated care: SA HealthPlus. BMJ. 2005;330:662–5.

70. Segal L, Dunt D, Day SE. Introducing coordinated care (2): evaluation of design features and implementation processes implications for a preferred health system reform model. Health Policy. 2004;69:215–28.

71. Gardner K, Sibthorpe B. Impediments to change in an Australian trial of coordinated care. Journal of Health Services Research & Policy. 2002;7:2–7.

72. Kodner DL. The quest for integrated systems of care for frail older persons. Aging Clinical and Experimental Research. 2002;14:307–13.

73. Gardner K, Sibthorpe B. Impediments to change in an Australian trial of coordinated care. Journal of Health Services Research & Policy. 2002;7:2–7.

74. Béland F, Hollander MJ. Integrated models of care delivery for the frail elderly: international perspectives. Gaceta Sanitaria. 2011;25:138–46.

75. Perkins D, Owen A, Cromwell D, Adamson L, Eagar K, Quinset K, et al. The Illawarra Coordinated Care Trial: better outcomes with existing resources? Australian Health Review. 2001;24:172.

76. PricewaterhouseCoopers (Australia), Australia, Department of Health and Ageing. The national evaluation of the second round of coordinated care trials: coordination of care and efficiency of healthcare : lessons from the second round of Australian coordinated care trials : executive summary and findings. Canberra: Dept. of Health and Ageing; 2007.

77. Swerissen H. Toward greater integration of the health system. Australian Health Review. 2002;25:88.

78. Hebert R, Raiche M, Dubois M-F, Gueye NR, Dubuc N, Tousignant M, et al. Impact of PRISMA, a Coordination-Type Integrated Service Delivery System for Frail Older People in Quebec (Canada): A Quasi-experimental Study. The Journals of Gerontology Series B: Psychological Sciences and Social Sciences. 2010;65B:107–18.

79. Office of Evaluation Health Canada. Evaluation of the Innovation Strategy 2009-2010 to 2013-2014 [Internet]. Canada: Public Health Agency of Canada; 2015. Available from: http://www.phac-aspc.gc.ca/about_apropos/evaluation/reports-rapports/2014-2015/eis-sie/assets/pdf/eis-sie-eng.pdf

80. Godden S, McCoy D, Pollock A. Policy on the rebound: trends and causes of delayed discharges in the NHS. Journal of the Royal Society of Medicine. 2009;102:22–8.

81. Kodner DL. Whole-system approaches to health and social care partnerships for the frail elderly: an exploration of North American models and lessons. Health and Social Care in the Community. 2006;14:384–90.

82. Hébert R, Durand PJ, Dubuc N, Tourigny A. PRISMA: a new model of integrated service delivery for the frail older people in Canada. International Journal of Integrated Care [Internet]. 2003 [cited 2019 Mar 15];3. Available from: http://www.ijic.org/article/10.5334/ijic.73/

83. Béland F, Hollander MJ. Integrated models of care delivery for the frail elderly: international perspectives. Gaceta Sanitaria. 2011;25:138–46.

84. Bergman H, Béland F, Lebel P, Contandriopoulos A-P, Tousignant P, Brunelle Y. Care for Canada’s frail elderly population: Fragmentation or integration. CAN MED ASSOC J. 1997;157:1116–21.

85. Health Authorities, England. The Community Care (Delayed Discharges etc.) Act 2003- Guidance for Implementation [Internet]. UK: Department for Health; 2003. Available from: https://webarchive.nationalarchives.gov.uk/20120503190444/http://www.dh.gov.uk/prod_consum_dh/groups/dh_digitalassets/@dh/@en/documents/digitalasset/dh_4064939.pdf

86. Roll J, Wright K. The Community Care (Delayed Discharges etc) Bill. London: House of Commons Library; 2002.

87. Henwood M. Effective partnership working: a case study of hospital discharge. Health and Social Care in the Community. 2006;14:400–7.

88. Lewis R, Glasby J. Delayed discharge from mental health hospitals: results of an English postal survey. Health and Social Care in the Community. 2006;14:225–30.

89. Wanless D. Securing our Future Health: Taking a Long-Term View. :179.

90. Rantala R, Larsen M, Gulis G, Koudenburg O, Armada F. Intersectoral action on health in urban settings – the experience of Varde Municipality, Denmark, 2007-201. :1.

91. Peck, David Towell, Pauline Gullive E. The meanings of culture in health and social care: a case study of the combined Trust in Somerset. Journal of Interprofessional Care. 2001;15:319–27.

92. Ball R, Forbes T, Parris M, Forsyth L. The Evaluation of Partnership Working in the Delivery of Health and Social Care. Public Policy and Administration. 2010;25:387–407.

93. Peck E, Gulliver P, Towell D. Governance BlackwellScience,Ltd of partnership between health and social services: the experience in Somerset. Health and Social Care in the Community. 2002;8.

94. Glasby J, Peck E. Care trusts: partnership working in action. Abingdon, U.K.: Radcliffe Medical Press; 2004.

95. Gulliver P, Peck E, Towell D. Evaluation of the Integration of Health and Social Services in Somerset: Part 2 ‐ Lessons for Other Localities. Journal of Integrated Care. 2002;10:33–8.

96. Newman M, EPPI-Centre. Commissioning in health, education and social care: models, research bibliography and in-depth review of joint commissioning between health and social care agencies : technical report. London: EPPI-Centre, Social Science Research Unit, Institute of Education, University of London; 2012.

97. Peck E, Perri 6, Gulliver P, Towell D. Why do we keep on meeting like this? The board as ritual in health and social care. Health Services Management Research. 2004;17:100–9.

98. Ball R, Forbes T, Parris M, Forsyth L. The Evaluation of Partnership Working in the Delivery of Health and Social Care. Public Policy and Administration. 2010;25:387–407.

99. Board GG. COMMUNITY HEALTH AND CARE PARTNERSHIPS WITH GLASGOW CITY COUNCIL. Glasgow: NHS Greater Glasgow and Clyde; 2010.

100. Windle DK. National Evaluation of Partnerships for Older People Projects: Final Report [Internet]. 2009 p. 302. Available from: https://www.pssru.ac.uk/pub/dp2700.pdf

101. North East Lincolnshire Clinical Commissioning Group. NORTH EAST LINCOLNSHIRE CCG Strategic Plan 2014 – 2019 [Internet]. North East Lincolnshire: NHS; 2014. Available from: http://www.northeastlincolnshireccg.nhs.uk/data/uploads/publications/nelccg-narrative-strategic-plan-dec-14.pdf

102. Ham C, Smith J. Removing the policy barriers to integrated care in England. 2010;20.

103. Goodwin N, Smith J. The Evidence Base for Integrated Care . The King’s Fund and the Nuffield. Trust; 2011.

104. Ferguson R, Craig M, Craig F, Biggar J, Walker A, Stuart A. Evaluation of Integrated Resource Framework Test Sites. The Scottish Government. 2012;

105. Øvretveit J, Hansson J, Brommels M. An integrated health and social care organisation in Sweden: Creation and structure of a unique local public health and social care system. Health Policy. 2010;97:113–21.

106. Lesnick T, Musliner M, Foley K, Weissert W. Cost Savings from Home and Community-Based Services: Arizona’s Capitated Medicaid Long-Term Care Program [Internet]. EBSCO Publishing; 2003. Available from: file:///C:/Users/Admin/Downloads/Lavanya%20References/Arizona%20Long%20Term%20Care%20System/ContentServer.pdf

107. Meyer H. A New Care Paradigm Slashes Hospital Use And Nursing Home Stays For The Elderly And The Physically And Mentally Disabled. Health Affairs. 2011;30:412–5.

108. Master RJ, Eng C. Integrating Acute And Long-Term Care For High-Cost Populations. Health Affairs. 2001;20:161–72.

109. Master RJ. Massachusetts Medicaid and the Community Medical Alliance: A New Approach to Contracting and Care Delivery for Medicaid-Eligible Populations with AIDS and Severe Physical Disability. The American Journal of Managed Care. 1998;4:90–8.

110. Lynch M, Estes CL, Hernandez M. Chronic Care Initiatives for the Elderly: Can They Bridge the Gerontology-Medicine Gap? Journal of Applied Gerontology. 2005;24:108–24.

111. Ryan J, Super N. Dually Eligible for Medicare and Medicaid: Two for One or Double Jeopardy? NHPF Issue Brief. 2003;24.

112. Saucier P, Fralich J. Financing and Payment Issues in Rural Long-Term Care Integration. Journal of Applied Gerontology. 2001;20:409–25.

113. Kane RL, Homyak P, Bershadsky B, Flood S, Zhang H. Patterns of Utilization for the Minnesota Senior Health Options Program: MSHO UTILIZATION PATTERNS. Journal of the American Geriatrics Society. 2004;52:2039–44.

114. Kane RL, Homyak P, Bershadsky B, Lum Y-S, Siadaty MS. Outcomes of Managed Care of Dually Eligible Older Persons. The Gerontologist. 2003;43:165–74.

115. Kane RL, Weiner A, Homyak P, Bershadsky B. The Minnesota Senior Health Options Program: An Early Effort at Integrating Care for the Dually Eligible. The Journals of Gerontology Series A: Biological Sciences and Medical Sciences. 2001;56:M559–66.

116. Boult C, Pacala JT. Integrating Healthcare for Older Populations. The American Journal of Managed Care. 1999;5:45–52.

117. Johri M, Beland F, Bergman H. International experiments in integrated care for the elderly: a synthesis of the evidence. International Journal of Geriatric Psychiatry. 2003;18:222–35.

118. Brummel-Smith K, Newcomer R, Harrington C, Kane R. GERIATRICS IN MANAGED CARE: Implementing the Second Generation Social Health Maintenance Organization. Journal of the American Geriatrics Society. 2000;48:829–34.

119. Newcomer R, Harrington C, Kane R. Challenges and Accomplishments of the Second-Generation Social Health Maintenance Organization. The Gerontologist. 2002;42:843–52.

120. Thomas KE, Gassoumis ZD, Wilber KH. Conversion Diversion: Participation in a Social HMO Reduces the Likelihood of Converting From Short-Stay to Long-Stay Nursing Facility Placement. Journal of the American Medical Directors Association. 2010;11:333–7.

121. Thompson TG. Evaluation Results for the Social/Health Maintenance. :94.

122. Kodner DL, Kyriacou CK. Fully integrated care for frail elderly: two American models. International Journal of Integrated Care [Internet]. 2000 [cited 2019 Mar 15];1. Available from: http://www.ijic.org/article/10.5334/ijic.11/

123. Leutz W, Nonnenkamp L, Dickinson L, Brody K. Utilization and costs of home-based and community-based care within a social HMO: trends over an 18-year period. International Journal of Integrated Care [Internet]. 2005 [cited 2019 Mar 15];5. Available from: http://www.ijic.org/article/10.5334/ijic.143/

124. Jha AK, Kizer KW. Effect of the Transformation of the Veterans Affairs Health Care System on the Quality of Care. The New England Journal of Medicine. 2003;10.

125. Kizer KW, Dudley RA. Extreme Makeover: Transformation of the Veterans Health Care System. Annual Review of Public Health. 2009;30:313–39.

126. Oliver A. The Veterans Health Administration: An American Success Story?: *Veterans Health Administration: American Success Story* ? Milbank Quarterly. 2007;85:5–35.

127. Oliver A. Public-sector health-care reforms that work? A case study of the US Veterans Health Administration. The Lancet. 2008;371:1211–3.

128. Baker G R, MacIntosh-Murray A, Porcellato C, Dionne L, Stelmacovich K, Born K. High Performing Healthcare Systems: Delivering Quality by Design. Toronto: Longwoods Publishing; 2008.

129. Goldman HH, Morrissey JP, Rosenheck RA, Cocozza J, Blasinsky M, Randolph F, et al. Lessons From the Evaluation of the ACCESS Program. Psychiatric Services. 2002;53:967–9.

130. Goldman HH, Morrissey JP, Rosenheck RA, Cocozza J, Blasinsky M, Randolph F, et al. Service Systems Integration and Outcomes for Mentally Ill Homeless. Persons in the ACCESS Program PSYCHIATRIC SERVICES. 2002. p. 959–66.

131. Rosenheck R, Morrissey J, Lam J, Calloway M, Stolar M, Johnsen M. Service Delivery and Community: Social Capital, Service Systems Integration, and Outcomes Among Homeless Persons with Severe Mental Illness. Health Services Research. 2001;36:691–710.

132. Rosenheck R, Morrissey J, Lam J, Calloway M, Johnsen M, Goldman H, et al. Service system integration, access to services, and housing outcomes in a program for homeless persons with severe mental illness. American Journal of Public Health. 1998;88:1610–5.

133. Randolph F, Blasinsky M, Morrissey JP, Rosenheck RA, Cocozza J, Goldman HH, et al. Overview of the ACCESS Program. Psychiatric Services. 2002;53:945–8.

134. Welsh WN, Knudsen HK, Knight K, Ducharme L, Pankow J, Urbine T. Effects of an Organizational Linkage Intervention on Inter- Organizational Service Coordination Between Probation/Parole Agencies and. Community Treatment Providers Adm Policy Ment Health. 2015;

135. Robison W, Chelala C, Freund P, Graybill E. A Healthy Child in a Healthy School Environment - A look at CHANGES: Program in Zambia. USA: Creative Associates International Inc; 2004.

136. Freund P, Graybill E, Keith N. Health and Education Working Together - A Case Study of a Successful School Health and Nutrition Model [Internet]. Zambia: CHANGES Program; 2005. Available from: https://41pylqn86jp37e3n04us8vqq-wpengine.netdna-ssl.com/wp-content/uploads/2014/05/ZambiaHealth_EdFINAL.pdf

137. Education M o. National School Health and Nutrition Policy. Lusaka: Republic of Zambia; 2006.

138. Road Safety Partnership Grant, 2007-09 Schemes: Headline Impact Report. :20.

139. King B, Surtees-Goodall S, Jeanes M. The Road Safety Partnership Grant Programme Summary Report of Impact of Round Two Projects and Progress. 2011.

140. Transport D f. Review of the Road Safety Partnership. Grant Scheme. Department for Transport; 2009.

141. Kickbusch I, Buckett K, Government of South Australia. Department of Health. Implementing health in all policies: adelaide 2010. Rundle Mall, SA: Government of South Australia. Department of Health; 2010.

142. Delany T, Lawless A, Baum F, Popay J, Jones L, McDermott D, et al. Health in All Policies in South Australia: What has supported early implementation? Health Promotion International. 2016;31:888–98.

143. Delany T, Harris P, Williams C, Harris E, Baum F, Lawless A, et al. Health Impact Assessment in New South Wales & Health in All Policies in South Australia: differences, similarities and connections. BMC Public Health [Internet]. 2014 [cited 2019 Mar 15];14. Available from: http://bmcpublichealth.biomedcentral.com/articles/10.1186/1471-2458-14-699

144. Australia G o. The South Australian approach to. Health in All Policies: background and guide to practise. 2011.

145. Eyk H v, Harris E, Baum F, Delany-Crowe T, Lawless A, MacDougall C. Health in All Policies in South Australia—Did It Promote and Enact an Equity Perspective. International Journal of Environmental Research and Public Health. 2017;14.

146. Hainsworth G, Zilhao I. From inception to large scale: The Geração Biz Programme in Mozambique. WHO. 2009;44.

147. Osman NB, Zilhao I. Sexual and reproductive rights of young people: Understanding and meeting the need. Increasing access and demand to sexual and reproductive health and rights among adolescents in Mozambique. International Journal of Gynecology and Obstetrics. 2009;107:S63.

148. Chandra-Mouli V, Gibbs S, Badiani R, Quinhas F, Svanemyr J. Programa Geração Biz, Mozambique: how did this adolescent health initiative grow from a pilot to a national programme, and what did it achieve? Reproductive Health [Internet]. 2015 [cited 2019 Mar 15];12. Available from: https://reproductive-health-journal.biomedcentral.com/articles/10.1186/1742-4755-12-12

149. Garg R, Karpati A, Leighton J, Perrin M, Shah M. Asthma Facts: Second Edition [Internet]. NYC: NYC Department of Health and Mental Hygiene; 2003. Available from: https://www1.nyc.gov/assets/doh/downloads/pdf/asthma/facts.pdf

150. Musuva RM, Matey E, Masaku J, Odhiambo G, Mwende F, Thuita I, et al. Lessons from implementing mass drug administration for soil transmitted helminths among pre-school aged children during school based deworming program at the Kenyan coast. BMC Public Health [Internet]. 2017 [cited 2019 Mar 15];17. Available from: http://bmcpublichealth.biomedcentral.com/articles/10.1186/s12889-017-4481-7

151. Okemo M, Sharif S. Kenya National School-Based Deworming Programme Year 1 (2012-2013) Results [Internet]. Ministry of Education, Science and Technology and Ministry of Health; 2012. Available from: file:///C:/Users/Admin/Downloads/Kenya_National_SchoolBased_Deworming_Programme_Year1_evaluation%20(2).pdf

152. Rotich L, Maina W. Kenya National School-Based Deworming Programme Year 2 Report (April 2013 - March 2014) [Internet]. Kenya: Ministry of Education, Science and Technology and Ministry of Health; 2013. Available from: file:///C:/Users/Admin/Downloads/Kenya_National_SchoolBased_Deworming_Programme_Year2_evaluation.pdf

153. Rotich L, Kioko J. Kenya National School-Based Deworming Programme Year 3 (2014-2015) Results [Internet]. Ministry of Education, Science and Technology and Ministry of Health; 2014. Available from: https://static1.squarespace.com/static/546f9316e4b0ced8102e4c74/t/583c5950414fb50504d8a8b4/1480350036170/NSBDP+Y3+Results+Booklet.pdf

154. Mwandawiro CS, Nikolay B, Kihara JH, Ozier O, Mukoko DA, Mwanje MT, et al. Monitoring and evaluating the impact of national school-based deworming in Kenya: study design and baseline results. Parasites & Vectors. 2013;6:198.

155. Nelson G, Prilleltensky I, Laurendeau M-C, Powell B. The prevention of mental health problems in Canada: A survey of provincial policies, structures, and programs. Canadian Psychology/Psychologie canadienne. 1996;37:161–72.

156. Gakh M. Law, the Health in all Policies Approach, and Cross-Sector Collaboration. Public Health Reports. 2015;130:96–100.

157. Officer G o. Mayors Council on Physical Fitness Health and Nutrition Establishment Act of 2011. Council of the District of Columbia. New York; 2011.

158. Heymann J, Hertzman C, Barer M, Evans R. Healthier Societies: From Analysis to Action. Oxford: Oxford University Press; 2006.

159. Clary A, Riley T. Pooling and Braiding Funds for Health-Related Social Needs: Lessons from Virginia’s Children’s Services Act. National Academy for State Health Policy; 2016.

160. Eyles J, Brimacombe M, Chaulk P, Stoddart G, Pranger T, Moase O. What determines health? To where should we shift resources? Attitudes towards the determinants of health among multiple stakeholder groups in Prince Edward Island, Canada. Social Science. 2001;9.

161. Scott AM, Li J, Oyewole-Eletu S, Nguyen HQ, Gass B, Hirschman KB, et al. Understanding Facilitators and Barriers to Care Transitions: Insights from Project ACHIEVE Site Visits. Joint Commission Journal on Quality and Patient Safety. 2017;43:433–47.

162. Scott R, Boyd R. Interagency Performance Targets A Case Study of New Zealand’s Results Programme. IBM Centre for The Business of Government. 2017.

163. Treasury T. Cross-Agency Funding Framework Guidance for funding cross-agency initiatives. New: Zealand Government; 2015.

164. Clary A, Riley T. Braiding & Blending Funding Streams to Meet the Health-Related Social Needs of Low-Income Persons: Considerations for State Health Policymakers. National Academy for State Health Policy. 2016;20.

165. Massachusetts Health Policy Commission - Health Care Innovation Investment Program (Round 1): Challenge Descriptions. Massachusetts Health Policy Commission; 2012.

166. Clary A, Riley T. Braiding & Blending Funding Streams to Meet the Health-Related Social Needs of Low-Income Persons: Considerations for State Health Policymakers. National Academy for State Health Policy. 2016;20.

167. Forum I (n. d. Maximizing Limited Resources Through Cross-Sector Partnerships [Internet]. Available from: http://www.astho.org/uploadedFiles/Programs/Health_Systems_Transformation/Primary_Care_and_Public_Health_Integration/Maximizing-Limited-Resources-Through-Cross-Sector-Partnerships.pdf

168. Clary A, Riley T. Pooling and Braiding Funds for Health-Related Social Needs: Lessons from Virginia’s Children’s Services Act. National Academy for State Health Policy; 2016.

169. User Guide for the Children’s Services Act (CSA User Guide). Office of Children’s Services; 2016.

170. Flaming D, Lee S, Burns P, Sumner G. Getting Home: Outcomes from Housing High Cost Homeless Hospital Patients. Conrad N. Hilton Foundation; 2013.

171. Management OP. A Joint Evaluation Uganda’s Plan for the Modernisation of Agriculture. Ministry of Foreign Affairs Denmark. 2005;

172. Gandhi G. Charting the evolution of approaches employed by the Global Alliance for Vaccines and Immunizations (GAVI) to address inequities in access to immunization: A systematic qualitative review of GAVI policies, strategies and resource allocation mechanisms through an equity lens (1999-2014). BMC Public Health. 2015;15.

173. A Joint Evaluation of Uganda’s Plan for the Modernisation of Agriculture [Internet]. Denmark: Ministry of Foreign Affairs Denmark; 2005. Available from: http://www.netpublikationer.dk/um/6207/pdf/200506PMAevaluation.pdf

174. IVAR. Working in place: Collaborative funding in practice - York Pathways: Supporting individuals experiencing mental distress [Internet]. London: Institute for Voluntary Action Research; 2017. Available from: https://www.ivar.org.uk/wp-content/uploads/2017/10/IVAR017-Working-in-Place-Report_York_Digital_FINAL.pdf

175. Wong K, Ellingworth D, Kinsella R, Lowthian J, Meadows L. Transition to Adulthood Pathway Programme Evaluation - First Interim Report [Internet]. Hallam Centre for Community Justice Sheffield Hallam University; 2016. Available from: https://www.t2a.org.uk/wp-content/uploads/2016/05/T2A-Interim-Report-1-Final-2.pdf

176. Wong K, Kinsella R, Bamonte J, Meadows L. T2A FINAL PROCESS EVALUATION REPORT. :50.

177. Batley R, Cabral L, Souza C. Sector Wide Approaches in Brazil: Features, drivers and emerging lessons -. :80.

178. Persaud A. Integrated Planning for Education and Development. Eur J Educ [Internet]. 2017;52. Available from: https://onlinelibrary.wiley.com/doi/pdf/10.1111/ejed.12233

179. Burdescu R, Parel C. Brazil: Designing a New Loan Prototype to Meet Client Needs. World Bank; 2006.

180. Unit F a. INTERNATIONAL BANK FOR RECONSTRUCTION AND DEVELOPMENT PROGRAM APPRAISAL DOCUMENT ON A PROPOSED LOAN. THE AMOUNT OF US$350 MILLION TO THE STATE OF CEARÁ, BRAZIL WITH THE GUARANTEE OF THE FEDERATIVE REPUBLIC OF BRAZIL TO STRENGTHEN SERVICE DELIVERY. FOR G. World Bank; 2013.

181. <IMPLEMENTATION COMPLETION AND RESULTS REPORT (IBRD-73210) ON A LOAN IN THE AMOUNT OF US$ 149.750 MILLION TO THE STATE OF CEARÁ, BRAZIL FOR A CEARÁ MULTI-SECTOR INCLUSION DEVELOPMENT PROJECT.pdf>.

182. Worton SK, Caplan R, Nelson G, Pancer SM, Loomis C, Peters RDeV, et al. Better Beginnings, Better Futures: Theory, research, and knowledge transfer of a community-based initiative for children and families. Psychosocial Intervention. 2014;23:135–43.

183. MSPAS) MO (n. d. INTERSECTORIAL EXPERIENCE IN THE EMPOWERMENT OF ADOLESCENT GIRLS REPUBLIC OF EL. SALVADOR, CENTRAL: AMERICA;

184. Peters RD, Bradshaw AJ, Petrunka K, Nelson G, Herry Y, Craig WM. THE BETTER BEGINNINGS, BETTER FUTURES PROJECT: FINDINGS FROM GRADE 3 TO. GRADE 9 Monographs of the Society for Research in Child Development. 2010;75:1–176.

185. McDaid D, Wilson E, Knapp M. Barriers and facilitators to commissioning cost-effective services for promotion of mental health and wellbeing and prevention of mental ill-health [Internet]. 2017 [cited 2019 May 5]. Available from: https://www.gov.uk/government/organisations/public-health-england

186. Suhonen M, Paasivaara L. Project work in Finnish KASTE projects. :17.

187. Larsen M, Rantala R, Koudenburg OA, Gulis G. Intersectoral action for health: The experience of a Danish municipality. Scandinavian Journal of Public Health. 2014;42:649–57.

188. Vuorenkoski L. New national development programme. National Institute for Health and Welfare (THL; 2008.

189. Kallinen S. National Development Plan for Social Welfare and Healthcare (Kaste Programme) 2012-2015 Final report. Ministry of Social Affairs and Health. 2016;

190. Health M o. The National Development Programme for Social Welfare and Health. Care THE KASTE PROGRAMME. 2012;2012.

191. Kehittämis K, Kaste O. Sosiaali- ja terveydenhuollon. :140.

192. Rantala R, Larsen M, Gulis G, Koudenburg O, Armada F. Intersectoral action on health in urban settings – the experience of Varde Municipality, Denmark, 2007-201. :1.

193. Larsen M, Rantala R, Koudenburg OA, Gulis G. Intersectoral action for health: The experience of a Danish municipality. Scandinavian Journal of Public Health. 2014;42:649–57.

194. Public Health Agency of Canada, Health Systems Knowledge Network, EQUINET (Organization). Crossing sectors: experiences in intersectoral action, public policy and health [Internet]. Ottawa, Ont.: Public Health Agency of Canada; 2007 [cited 2019 Mar 15]. Available from: http://www.phac-aspc.gc.ca/publicat/2007/cro-sec/pdf/cro-sec_e.pdf

195. World Health Organization, Public Health Agency of Canada, editors. Health equity through intersectoral action: an analysis of 18 country case studies. Ottawa: Public Health Agency of Canada; 2008.

196. Sharma B, Nam E. A Healthy City Project: A Case Study of Wonju City, South Korea and its Relevance to the Cities in Nepal. Journal of Gandaki Medical College-Nepal. 2017;10:34–42.

197. Taylor M. Bristol Impact Fund: Coordinating council grant streams - Case Study [Internet]. Institute for Voluntary Action Research; 2017. Available from: https://www.ivar.org.uk/publication/bristol-impact-fund-coordinating-council-grant-streams/

198. Sharma B, Nam E. A Healthy City Project: A Case Study of Wonju City, South Korea and its Relevance to the Cities in Nepal. Journal of Gandaki Medical College-Nepal. 2017;10:34–42.
