# Supplementary material for: Financing intersectoral action for health: a systematic review of co-financing models
Source: Global Health. 2019 Dec 18;15:86. doi: 10.1186/s12992-019-0513-7 (PMC6918645; doi:10.1186/s12992-019-0513-7)
Supplement: Supplementary file 2 — Additional file 2. Search String used in 3 searches: Ovid Search terms for EconLit, MEDLINE, EMBASE, PsycINFO, HMIC, Global Health, Social Policy & Practise. Scopus, Open Grey [file 12992_2019_513_MOESM2_ESM.docx]

**Additional File 2: Sample Search Strategy**

1. *Ovid Search terms for EconLit, MEDLINE, EMBASE, PsycINFO, HMIC, Global Health, Social Policy & Practise*

*** for truncation**

**# for alternative letter in words**

**? for characters which are sometimes there sometimes not**

**adj# to find words within # words of each other**

**- searches both hyphenated and spaced words**

Across-sector* adj3 fund*

Across-sector* adj3 budget*

Across-sector* adj3 financ*

Across-sector* adj3 commission*

Across-sector* adj3 payment*

Align* adj3 fund*

Align* adj3 budget*

Align* adj3 financ*

Align* adj3 commission*

Align* adj3 payment*

Between-sector* adj3 fund*

Between-sector* adj3 budget*

Between-sector* adj3 financ*

Between-sector* adj3 commission*

Between-sector* adj3 payment*

Collaborat* adj3 fund*

Collaborat* adj3 budget*

Collaborat* adj3 financ*

Collaborat* adj3 commission*

Collaborat* adj3 payment*

Consolidate* adj3 fund*

Consolidate* adj3 budget*

Consolidate* adj3 financ*

Consolidate* adj3 commission*

Consolidate* adj3 payment*

Co-fund*

Co-budget*

Co-financ*

Co-commission*

Co-payment*

Cross-sector* adj3 fund*

Cross-sector* adj3 budget*

Cross-sector* adj3 financ*

Cross-sector* adj3 commission*

Cross-sector* adj3 payment*

Flexible adj3 fund*

Flexible adj3 budget*

Flexible adj3 financ*

Flexible adj3 commission*

Flexible adj3 payment*

Fund* adj3 intersector* adj3 collaborat*

Financ* adj3 intersector* adj3 collaborat*

Commission* adj3 intersector* adj3 collaborat*

Integrate* adj3 fund*

Integrate* adj3 budget*

Integrate* adj3 financ*

Integrate* adj3 commission*

Integrate* adj3 payment*

Intersector* adj3 fund*

Intersector* adj3 budget*

Intersector* adj3 financ*

Intersector* adj3 commission*

Intersector* adj3 payment*

Inter-sector* adj3 fund*

Inter-sector* adj3 budget*

Inter-sector* adj3 financ*

Inter-sector* adj3 commission*

Inter-sector* adj3 payment*

Inter-minist?r* adj3 fund*

Inter-minist?r* adj3 budget*

Inter-minist?r* adj3 financ*

Inter-minist?r* adj3 commission*

Inter-minist?r* adj3 payment*

Inter-organi#ation* adj3 fund*

Inter-organi#ation* adj3 budget*

Inter-organi#ation* adj3 financ*

Inter-organi#ation* adj3 commission*

Inter-organi#ation* adj3 payment*

Joint fund*

Joint budget*

Joint financ*

Joint commission*

Joint payment*

Joint resourc*

Multi*-sector* adj3 fund*

Multi*-sector* adj3 budget*

Multi*-sector* adj3 financ*

Multi*-sector* adj3 commission*

Multi*-sector* adj3 payment*

Multi*-minist?r* adj3 fund*

Multi*-minist?r* adj3 budget*

Multi*-minist?r* adj3 financ*

Multi*-minist?r* adj3 commission*

Multi*-minist?r* adj3 payment*

Partnership adj3 fund*

Partnership adj3 budget*

Partnership adj3 financ*

Partnership adj3 commission*

Partnership adj3 payment*

Pool* fund*

Pool* budget*

Pool* financ*

Pool* commission*

Pool* payment*

Pool* resourc*

Shar* fund*

Shar* budget*

Shar* financ*

Shar* commission*

Shar* payment*

Shar* resourc*

Fund* silo*

Budget* silo*

Financ* silo*

Commission* silo*

Payment* silo*

Resourc* silo*

Across-sector adj3 resourc* adj3 pool*

Between-sector adj3 resourc* adj3 pool*

Cross-sectoral adj3 resourc* adj3 pool*

Multi*-sector* adj3 resourc* adj3 pool*

Multi*-minist?r* adj3 resourc* adj3 pool*

Inter-sector* adj3 resourc* adj3 pool*

Intersector* adj3 resourc* adj3 pool*

Inter-minist?r* adj3 resourc* adj3 pool*

Inter-organi#ation* adj3 resourc* adj3 pool*

Budget adj3 shar*

Budget partner*

Blend* Financ*

Co-benefit*

Cofinanc*

Cofund*

Copayment*

Join* government

Joined-up government

Whole-of-government

Promotion fund*

**AND**

Across-sector*

Between-sector*

Cross-sector*

Inter-organi#ation*

Inter-minist?r*

Multi*-sector*

Multi*-minist?r*

1. *Search terms for SCOPUS*

**“ ” for phrases**

*** for truncation**

**? for alternative letter in words**

**N# to find words within # words of each other**

**W# to find words within # words of each other in that order**

“Across-sector* W/3 fund*” OR

“Across-sector* W/3 budget*” OR

“Across-sector* W/3 financ*” OR

“Across-sector* W/3 commission*” OR

“Across-sector* W/3 payment*” OR

“Across sector* W/3 fund*” OR

“Across sector* W/3 budget*” OR

“Across sector* W/3 financ*” OR

“Across sector* W/3 commission*” OR

“Across sector* W/3 payment*” OR

“Align* W/3 fund*” OR

“Align* W/3 budget*” OR

“Align* W/3 financ*” OR

“Align* W/3 commission*” OR

“Align* W/3 payment*” OR

“Between-sector* W/3 fund*” OR

“Between-sector* W/3 budget*” OR

“Between-sector* W/3 financ*” OR

“Between-sector* W/3 commission*” OR

“Between-sector* W/3 payment*” OR

“Between sector* W/3 fund*” OR

“Between sector* W/3 budget*” OR

“Between sector* W/3 financ*” OR

“Between sector* W/3 commission*” OR

“Between sector* W/3 payment*” OR

“Collaborat* W/3 fund*” OR

“Collaborat* W/3 budget*” OR

“Collaborat* W/3 financ*” OR

“Collaborat* W/3 commission*” OR

“Collaborat* W/3 payment*” OR

“Consolidate* W/3 fund*” OR

“Consolidate* W/3 budget*” OR

“Consolidate* W/3 financ*” OR

“Consolidate* W/3 commission*” OR

“Consolidate* W/3 payment*” OR

“Co-fund*” OR

“Co-budget*” OR

“Co-financ*” OR

“Co-commission*” OR

“Co-payment*” OR

“Cross-sector* W/3 fund*” OR

“Cross-sector* W/3 budget*” OR

“Cross-sector* W/3 financ*” OR

“Cross-sector* W/3 commission*” OR

“Cross sector* W/3 payment*” OR

“Cross sector* W/3 fund*” OR

“Cross sector* W/3 budget*” OR

“Cross sector* W/3 financ*” OR

“Cross sector* W/3 commission*” OR

“Cross sector* W/3 payment*” OR

“Flexible W/3 fund*” OR

“Flexible W/3 budget*” OR

“Flexible W/3 financ*” OR

“Flexible W/3 commission*” OR

“Flexible W/3 payment*” OR

“Fund* W/3 intersector* W/3 collaborat*” OR

“Financ* W/3 intersector* W/3 collaborat*” OR

“Commission* W/3 intersector* W/3 collaborat*” OR

“Integrate* W/3 fund*” OR

“Integrate* W/3 budget*” OR

“Integrate* W/3 financ*” OR

“Integrate* W/3 commission*” OR

“Integrate* W/3 payment*” OR

“Intersector* W/3 fund*” OR

“Intersector* W/3 budget*” OR

“Intersector* W/3 financ*” OR

“Intersector* W/3 commission*” OR

“Intersector* W/3 payment*” OR

“Inter-sector* W/3 fund*” OR

“Inter-sector* W/3 budget*” OR

“Inter-sector* W/3 financ*” OR

“Inter-sector* W/3 commission*” OR

“Inter-sector* W/3 payment*” OR

“Inter sector* W/3 fund*” OR

“Inter sector* W/3 budget*” OR

“Inter sector* W/3 financ*” OR

“Inter sector* W/3 commission*” OR

“Inter sector* W/3 payment*” OR

“Inter-minister* W/3 fund*” OR

“Inter-minister* W/3 budget*” OR

“Inter-minister* W/3 financ*” OR

“Inter-minister* W/3 commission*” OR

“Inter-minister* W/3 payment*” OR

“Inter minister* W/3 fund*” OR

“Inter minister* W/3 budget*” OR

“Inter minister* W/3 financ*” OR

“Inter minister* W/3 commission*” OR

“Inter minister* W/3 payment*” OR

“Inter-ministr* W/3 fund*” OR

“Inter-ministr* W/3 budget*” OR

“Inter-ministr* W/3 financ*” OR

“Inter-ministr* W/3 commission*” OR

“Inter-ministr* W/3 payment*” OR

“Inter ministr* W/3 fund*” OR

“Inter ministr* W/3 budget*” OR

“Inter ministr* W/3 financ*” OR

“Inter ministr* W/3 commission*” OR

“Inter ministr* W/3 payment*” OR

“Inter-organi?ation* W/3 fund*” OR

“Inter-organi?ation* W/3 budget*” OR

“Inter-organi?ation* W/3 financ*” OR

“Inter-organi?ation* W/3 commission*” OR

“Inter-organi?ation* W/3 payment*” OR

“Inter organi?ation* W/3 fund*” OR

“Inter organi?ation* W/3 budget*” OR

“Inter organi?ation* W/3 financ*” OR

“Inter organi?ation* W/3 commission*” OR

“Inter organi?ation* W/3 payment*” OR

“Joint fund*” OR

“Joint budget*” OR

“Joint financ*” OR

“Joint commission*” OR

“Joint payment*” OR

“Joint resourc*” OR

“Multi*-sector* W/3 fund*” OR

“Multi*-sector* W/3 budget*” OR

“Multi*-sector* W/3 financ*” OR

“Multi*-sector* W/3 commission*” OR

“Multi*-sector* W/3 payment*” OR

“Multi* sector* W/3 fund*” OR

“Multi* sector* W/3 budget*” OR

“Multi* sector* W/3 financ*” OR

“Multi* sector* W/3 commission*” OR

“Multi* sector* W/3 payment*” OR

“Multi*-minister* W/3 fund*” OR

“Multi*-minister* W/3 budget*” OR

“Multi*-minister* W/3 financ*” OR

“Multi*-minister* W/3 commission*” OR

“Multi*-minister* W/3 payment*” OR

“Multi* minister* W/3 fund*” OR

“Multi* minister* W/3 budget*” OR

“Multi* minister* W/3 financ*” OR

“Multi* minister* W/3 commission*” OR

“Multi* minister* W/3 payment*” OR

“Multi*-ministr* W/3 fund*” OR

“Multi*-ministr* W/3 budget*” OR

“Multi*-ministr* W/3 financ*” OR

“Multi*-ministr* W/3 commission*” OR

“Multi*-ministr* W/3 payment*” OR

“Multi* ministr* W/3 fund*” OR

“Multi* ministr* W/3 budget*” OR

“Multi* ministr* W/3 financ*” OR

“Multi* ministr* W/3 commission*” OR

“Multi* ministr* W/3 payment*” OR

“Partnership W/3 fund*” OR

“Partnership W/3 budget*” OR

“Partnership W/3 financ*” OR

“Partnership W/3 commission*” OR

“Partnership W/3 payment*” OR

“Pool* fund*” OR

“Pool* budget*” OR

“Pool* financ*” OR

“Pool* commission*” OR

“Pool* payment*” OR

“Pool* resourc*” OR

“Shar* fund*” OR

“Shar* budget*” OR

“Shar* financ*” OR

“Shar* commission*” OR

“Shar* payment*” OR

“Shar* resourc*” OR

“Fund* silo*” OR

“Budget* silo*” OR

“Financ* silo*” OR

“Commission* silo*” OR

“Payment* silo*” OR

“Resourc* silo*” OR

“Across-sector W/3 resourc* W/3 pool*” OR

“Between-sector W/3 resourc* W/3 pool*” OR

“Cross-sectoral W/3 resourc* W/3 pool*” OR

“Multi*-sector* W/3 resourc* W/3 pool*” OR

“Multi*-minister* W/3 resourc* W/3 pool*” OR

“Multi*-ministr* W/3 resourc* W/3 pool*” OR

“Inter-sector* W/3 resourc* W/3 pool*” OR

“Across sector W/3 resourc* W/3 pool*” OR

“Between sector W/3 resourc* W/3 pool*” OR

“Cross sectoral W/3 resourc* W/3 pool*” OR

“Multi* sector* W/3 resourc* W/3 pool*” OR

“Multi* minister* W/3 resourc* W/3 pool*” OR

“Multi* ministr* W/3 resourc* W/3 pool*” OR

“Inter sector* W/3 resourc* W/3 pool*” OR

“Intersector* W/3 resourc* W/3 pool*” OR

“Inter-minister* W/3 resourc* W/3 pool*” OR

“Inter-ministr* W/3 resourc* W/3 pool*” OR

“Inter-organi?ation* N3 resourc* W/3 pool*” OR

“Inter minister* W/3 resourc* W/3 pool*” OR

“Inter ministr* W/3 resourc* W/3 pool*” OR

“Inter organi?ation* W/3 resourc* W/3 pool*” OR

“Budget W/3 3shar*” OR

“Budget partner*” OR

“Blend* financ*” OR

"Co-benefit*" OR

"Cofinanc*" OR

"Cofund*" OR

"Copayment*" OR

"Join* W/3 government" OR

"Joined-up W/3 government" OR

"Whole-of-government" OR

"Promotion W/3 fund*"

**AND**

“Across-sector*” OR

“Across sector*” OR

“Between-sector*” OR

“Between sector*” OR

“Cross-sector*” OR

“Cross sector*” OR

“Inter-organi?ation*” OR

“Inter organi?ation*” OR

“Inter-minister*” OR

“Inter minister*” OR

“Inter-ministr*” OR

“Inter ministr*” OR

“Multi*-sector*” OR

“Multi* sector*” OR

“Multi*-minister*” OR

“Multi* minister*” OR

“Multi*-ministr*” OR

“Multi* ministr*”

1. *Search terms for Open Grey*

Near means within 16 words of each other.

**“ ” for phrases**

*** for truncation**

"Across sector* NEAR fund*" OR

"Across sector* NEAR budget*" OR

"Across sector* NEAR financ*" OR

"Across sector* NEAR commission*" OR

"Across sector* NEAR payment*" OR

"Align* NEAR fund*" OR

"Align* NEAR budget*" OR

"Align* NEAR financ*" OR

"Align* NEAR commission*" OR

"Align* NEAR payment*" OR

"Between sector* NEAR fund*" OR

"Between sector* NEAR budget*" OR

"Between sector* NEAR financ*" OR

"Between sector* NEAR commission*" OR

"Between sector* NEAR payment*" OR

"Collaborat* NEAR fund*" OR

"Collaborat* NEAR budget*" OR

"Collaborat* NEAR financ*" OR

"Collaborat* NEAR commission*" OR

"Collaborat* NEAR payment*" OR

"Consolidate* NEAR fund*" OR

"Consolidate* NEAR budget*" OR

"Consolidate* NEAR financ*" OR

"Consolidate* NEAR commission*" OR

"Consolidate* NEAR payment*" OR

"Co-fund*" OR

"Co-budget*" OR

"Co-financ*" OR

"Co-commission*" OR

"Co-payment*" OR

"Cross sector* NEAR payment*" OR

"Cross sector* NEAR fund*" OR

"Cross sector* NEAR budget*" OR

"Cross sector* NEAR financ*" OR

"Cross sector* NEAR commission*" OR

"Cross sector* NEAR payment*" OR

"Flexible NEAR fund*" OR

"Flexible NEAR budget*" OR

"Flexible NEAR financ*" OR

"Flexible NEAR commission*" OR

"Flexible NEAR payment*" OR

"Fund* intersector* collaborat*" OR

"Financ* intersector* collaborat*" OR

"Commission* intersector* collaborat*" OR

"Integrate* NEAR fund*" OR

"Integrate* NEAR budget*" OR

"Integrate* NEAR financ*" OR

"Integrate* NEAR commission*" OR

"Integrate* NEAR payment*" OR

"Intersector* NEAR fund*" OR

"Intersector* NEAR budget*" OR

"Intersector* NEAR financ*" OR

"Intersector* NEAR commission*" OR

"Intersector* NEAR payment*" OR

"Inter sector* NEAR fund*" OR

"Inter sector* NEAR budget*" OR

"Inter sector* NEAR financ*" OR

" “Inter sector* NEAR commission*" OR

"Inter sector* NEAR payment*" OR

"Inter minister* NEAR fund*" OR

"Inter minister* NEAR budget*" OR

"Inter minister* NEAR financ*" OR

"Inter minister* NEAR commission*" OR

"Inter minister* NEAR payment*" OR

"Inter ministr* NEAR fund*" OR

"Inter ministr* NEAR budget*" OR

"Inter ministr* NEAR financ*" OR

"Inter ministr* NEAR commission*" OR

"Inter ministr* NEAR payment*" OR

"Inter organisation* NEAR fund*" OR

"Inter organisation* NEAR budget*" OR

"Inter organisation* NEAR financ*" OR

"Inter organisation* NEAR commission*" OR

"Inter organisation* NEAR payment*" OR

"Inter organization* NEAR fund*" OR

"Inter organization* NEAR budget*" OR

"Inter organization* NEAR financ*" OR

"Inter organization* NEAR commission*" OR

"Inter organization* NEAR payment*" OR

"Joint fund*" OR

"Joint budget*" OR

"Joint financ*" OR

"Joint commission*" OR

"Joint payment*" OR

"Joint resourc*" OR

"Multi* sector* NEAR fund*" OR

"Multi* sector* NEAR budget*" OR

"Multi* sector* NEAR financ*" OR

"Multi* sector* NEAR commission*" OR

"Multi* sector* NEAR payment*" OR

"Multi* minister* NEAR fund*" OR

"Multi* minister* NEAR budget*" OR

"Multi* minister* NEAR financ*" OR

"Multi* minister* NEAR commission*" OR

"Multi* minister* NEAR payment*" OR

"Multi* ministr* NEAR fund*" OR

"Multi* ministr* NEAR budget*" OR

"Multi* ministr* NEAR financ*" OR

"Multi* ministr* NEAR commission*" OR

"Multi* ministr* NEAR payment*" OR

"Partnership fund*" OR

"Partnership budget*" OR

"Partnership financ*" OR

"Partnership commission*" OR

"Partnership payment*" OR

"Pool* fund*" OR

"Pool* budget*" OR

"Pool* financ*" OR

"Pool* commission*" OR

"Pool* payment*" OR

"Pool* resourc*" OR

"Shar* fund*" OR

"Shar* budget*" OR

"Shar* financ*" OR

"Shar* commission*" OR

"Shar* payment*" OR

"Shar* resourc*" OR

"Fund* silo*" OR

"Budget* silo*" OR

"Financ* silo*" OR

"Commission* silo*" OR

"Payment* silo*" OR

"Resourc* silo*" OR

"Inter-sector* resourc* pool*" OR

"Across sector* resourc* pool*" OR

"Between sector* resourc* pool*" OR

"Cross sector* resourc* pool*" OR

"Multi* sector* resourc* pool*" OR

"Multi* minister* resourc* pool*" OR

"Multi* ministr* resourc* pool*" OR

"Inter sector* resourc* pool*" OR

"Intersector* resourc* pool*" OR

"Inter minister* resourc* pool*" OR

"Inter ministr* resourc* pool*" OR

"Inter organisation* resourc* pool*" OR

"Inter organization* resourc* pool*" OR

"Budget shar*" OR

"Budget partner*" OR

"Blend* financ*"
